# A constitutive modelling framework for applications to *in vivo* longitudinal data: Evaluation in a 60‐day head‐down bed rest study on arterial function

**DOI:** 10.1113/JP289863

**Published:** 2026-03-24

**Authors:** Alessandro Giudici, Karen Barchetti, Umit Gencer, Hakim Khettab, Elie Mousseaux, Carole Leguy, Tammo Delhaas, Rosa Maria Bruno, Bart Spronck, Pierre Boutouyrie

**Affiliations:** ^1^ Department of Biomedical Engineering, Cardiovascular Research Institute Maastricht (CARIM) Maastricht University Maastricht The Netherlands; ^2^ GROW Institute of Oncology and Reproduction Maastricht University Maastricht The Netherlands; ^3^ Université Paris Cité, INSERM U970, Paris Cardiovascular Research Centre – PARCC Paris France; ^4^ Hôpital Européen Georges Pompidou, Assistance Publique‐Hopitaux de Paris Pharmacology and Hypertension and Radiology Units Paris France; ^5^ Department of Cardiovascular Engineering, Institute of Applied Medical Engineering Helmholtz Institute, Medical Faculty RWTH Aachen University Aachen Germany; ^6^ Institute of Measurement Engineering and Sensor Technology University of Applied Sciences Ruhr West Mülheim an der Ruhr Germany; ^7^ Macquarie Medical School Faculty of Medicine, Health and Human Sciences Macquarie University, Macquarie Park Sydney NSW Australia

**Keywords:** arterial biomechanical homeostasis, arterial computational constitutive modelling, arterial remodelling, arterial stiffness, head‐down bed rest

## Abstract

**Abstract:**

Vascular cells continuously remodel the arterial wall (micro)structure in response to changes in their biomechanical/biochemical environment. Although the functional effects of arterial remodelling can be easily measured, assessing the underlying microstructural mechanisms is complex *in vivo*. Constitutive modelling is a computational technique that allows for linking whole‐organ function to tissue constituent‐level mechanics. However, the need for comprehensive biomechanical data for model parametrisation hampers its clinical applicability. In the present study, we propose a novel constitutive modelling framework that addresses this limitation by leveraging longitudinal acquisitions of pressure–diameter relationships at different arterial beds to aid model parametrisation. We applied our constitutive framework to data from a study on the effect of 60 days head‐down bed rest (HDBR) on arterial function, where pressure–diameter relationships of three arteries (carotid, femoral and popliteal) were measured at baseline, during HDBR (two time points) and during a 30‐day recovery (two time points). We modelled the arterial wall as a constrained mixture of elastin, collagen and vascular smooth muscle cells (VSMCs). The dimensionality of the parameterisation problem was reduced through assumptions on (i) the time evolution of the behaviour of individual constituents and (ii) consistency in intrinsic constituent mechanical properties across different arterial beds. Overall, the proposed framework captured well the *in vivo* data (*R* ^2^ = 0.89 ± 0.05). We identified increased VSMC contraction and microstructural re‐arrangement of collagen fibres as key adaptations to haemodynamic changes during HDBR, also resulting in reversible de‐stiffening of peripheral arteries. The proposed approach appears promising for disentangling microstructural mechanisms of arterial remodelling in clinical settings.

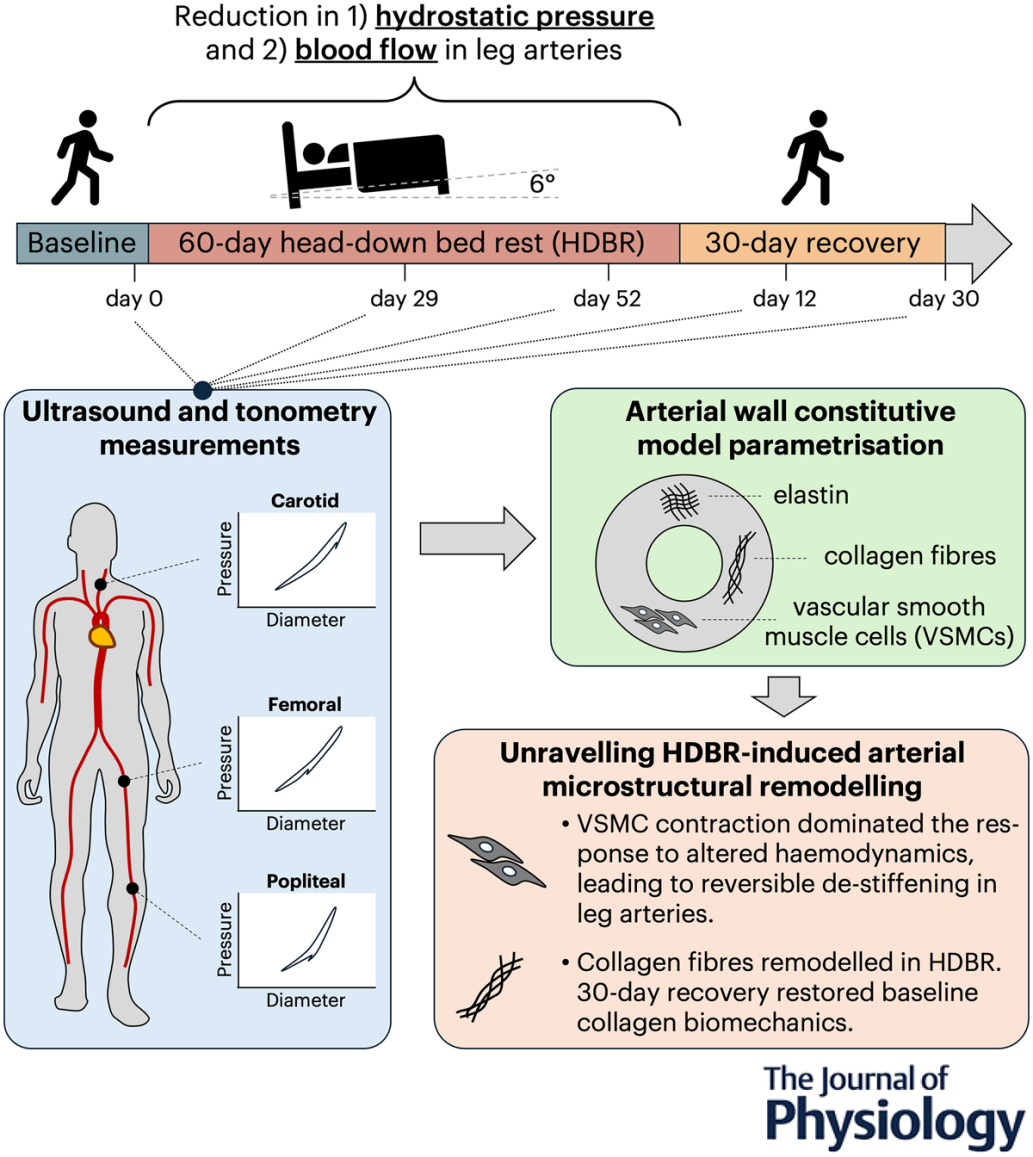

**Key points:**

- Constitutive modelling is a computational technique that links the macroscopic behaviour of arteries to the microstructure and mechanics of the constituents of their wall.
- Although constitutive modelling is used extensively on *ex vivo* data, the sparsity of biomechanical data that can be acquired *in vivo* hinders its applicability in clinical settings, where it could be instrumental in disentangling remodelling processes in ageing and disease.
- We propose a novel framework that leverages longitudinal acquisition of arterial waveforms at different arterial sites to aid in the parametrisation of comprehensive constitutive models.
- We exemplify the utility of our approach by teasing out the pivotal adaptation roles of vascular smooth muscle cell contraction and collagen microstructural remodelling in response to haemodynamic alterations resulting from prolonged head‐down bed rest.
- Our approach shows promise for the quantitative characterisation of arterial remodelling from non‐invasive *in vivo* data that can be easily measured in clinical settings.

## Graphical abstract

**Abstract figure legend** We proposed a computational constitutive modelling framework that leverages longitudinal acquisitions of haemodynamic waveforms at multiple arterial locations to achieve a biomechanical characterisation of their (micro)structural remodelling processes. We applied our approach to *in vivo* data from our previous study on the effect of simulated microgravity (i.e. 60‐day head‐down bed rest, HDBR) on arterial function, characterising the remodelling response to altered haemodynamics of the carotid, femoral and popliteal arteries. Our analysis provided a plausible microstructural interpretation of the vascular adaptation to HDBR of leg arteries, which was driven both by increased vasoconstriction during HDBR and by altered collagen biomechanics.

## Introduction

Function and (micro)structure are strongly interlinked in arteries. The composition and microstructural arrangement of constituents within the wall of arteries are crucial determinants of an artery's ability to store ventricular energy during systole in the form of elastic potential and then return it as elastic recoil in diastole. Elastin is a structural protein that provides compliance to the wall tissue at physiological pressures, whereas stiffer collagen fibres reinforce it at higher pressures, thus preserving tissue integrity (Burton, 1954; Cox, 1978; Roach & Burton, 1957). Notably, the arterial wall is not a static tissue in terms of (micro)structure and composition, but, in a process known as arterial remodelling, it continuously adapts to changes in biomechanical and biochemical conditions to maintain an optimal function (Humphrey, 2008; van Varik et al., 2012). Cells residing in the wall tissue (primarily endothelial cells, vascular smooth muscle cells (VSMCs) and fibroblasts) are pivotal actors in this process, as they can sense alterations in their biomechanical and biochemical environment and trigger long‐term remodelling processes that aim to preserve homeostasis (Humphrey, 2008). Furthermore, through contraction, VSMCs can also enact short‐term adaptations in arterial biomechanics (i.e., stress and stiffness) and structure (i.e., diameter and thickness). The disruption of arterial remodelling mechanisms is considered to be central to several disease processes (Jaminon et al., 2019; Spronck et al., 2021), which is why their characterisation is pivotal for devising strategies to prevent and counteract dysfunctional vascular ageing.

Characterising the arterial remodelling processes *in vivo* is complex. Indeed, although it is relatively easy to measure the structural and functional consequences of remodelling (e.g., by measuring changes in arterial stiffness as pulse wave velocity (PWV) and arterial structure as diameter and intima‐media thickness), we have no means by which to directly measure the underlying microstructural mechanisms in clinical settings. Constitutive modelling is a computational method that has been used to link tissue‐level biomechanics with microstructure and micromechanics at the constituents’ level (Giudici, Wilkinson, et al., 2021; Reesink & Spronck, 2018). It assumes that tissue‐level biomechanical behaviours are determined by the summed contributions of mechanically relevant microstructural constituents. Because of this, it constitutes a promising tool for indirectly inferring arterial remodelling processes from whole‐organ behaviours. However, its applicability to *in vivo* settings has been hindered by its need for extensive, biaxial biomechanical data for accurate model parameterisation. Indeed, to be physiologically relevant, a constitutive model should involve specific functions to describe not only the passive contributions of elastin and collagen, but also the active contraction of VSMCs, given that arteries probably exhibit a certain level of vascular tone *in vivo*. This implies a large number of model parameters (*n* = 8–14 depending on the model formulation) that need to be simultaneously fitted to sparse *in vivo* (uniaxial) biomechanical data, that is, a diastolic‐to‐systolic pressure–diameter relationship (Gade et al., 2021; Masson et al., 2011; Spronck et al., 2015). Notably, previous work showed that parameter estimation from such sparse *in vivo* data becomes problematic for models involving as few as four to five model parameters (Heusinkveld et al., 2018; Parikh et al., 2024). Therefore, devising strategies to facilitate constitutive model parameterisation on *in vivo* data remains a crucial step to bring constitutive modelling's potential to unravel vascular remodelling processes to clinical fruition.

In the present study, we propose a novel constitutive modelling framework that aims to specifically address model parametrisation issues for applications on *in vivo* data. The proposed framework combines two strategies to tackle the sparsity of biomechanical data acquired *in vivo*. First, rather than investigating the biomechanics of an artery at a single point in time, we observe the evolution of its behaviour over time (i.e., at different time points). This first strategy allows us to make evidence‐based assumptions to constrain the potential evolution over time of the microstructure and behaviour of individual constituents. Second, for each time point, we simultaneously evaluate the behaviour of different arterial beds rather than looking at a single artery. This second strategy allows us to assume consistency of the intrinsic properties of individual constituents across different arteries, which also contributes to reducing the dimensionality of the parametrisation problem. To develop this framework, we used data from the longitudinal study of Boutouyrie et al. (2022), who investigated the effect of 60‐day head‐down bed rest (HDBR), constituting a well‐established method for the simulation of microgravity on earth (Pandiarajan & Hargens, 2020), on arterial function. Therefore, the secondary aim of the present study is to disentangle the microstructural remodelling processes that arteries undergo in response to HDBR.

## Methods

### Kinematics

The carotid, femoral and popliteal arteries were modelled as incompressible thin‐walled cylinders. As done in previous *ex vivo* work (Bellini et al., 2014; Giudici, van der Laan, et al., 2023), we defined the deformation that arteries undergo *in vivo* during a cardiac cycle with respect to an *in vivo* reference configuration ($\kappa_{r}$). In the absence of torsion, any deformation from $\kappa_{r}$ is described by the deformation gradient:

(1) $$\begin{array}{c} F=\text{diag}\;\left[\frac{1}{\lambda_{\theta }\lambda_{z}},\lambda_{\theta },\lambda_{z}\right]=\text{diag}\left[\frac{R_{m}}{r_{m}\lambda_{z}},\frac{r_{m}}{R_{m}},\lambda_{z}\right] \end{array},$$

where $\lambda_{\theta }$ is the circumferential stretch, $\lambda_{z}$ is the axial stretch, and $\lambda_{r}=1/\left(\lambda_{\theta }\lambda_{z}\right)$ is the radial stretch expressed as a function of $\lambda_{\theta }$ and $\lambda_{z}$ because of the incompressibility constraint. $r_{m}$ and $R_{m}$ indicate the vessel mid‐wall radius in a generic deformed state and in $\kappa_{r}$, respectively. Notably, most arteries (except for the ascending aorta) maintain a fixed axial length *in vivo*, so that we can assume $\lambda_{z}=1$ for our application. This simplifies eqn (1) to:

(2) $$F\;=\;\mathrm{diag}\left[\frac{R_{m}}{r_{m}},\frac{r_{m}}{R_{m}},1\right].$$

### Constitutive modelling framework

The arterial wall was modelled as a constrained mixture of an isotropic elastin matrix reinforced by four families of collagen fibres and a single family of VSMCs. The behaviour of the arterial wall is governed by the strain energy density function (SEDF):

(3) $$\begin{array}{c} \Psi =\Phi_{e}\;\Psi_{e}+\Phi_{c}\Psi_{c}+\Phi_{\mathrm{VSMC}}\Psi_{\mathrm{VSMC}} \end{array},$$

where $\Phi_{i}$ is the volume fraction and $\Psi_{i}$ is the SEDF of the wall constituent i, with i∈[e,c,VSMC] for elastin, collagen, and VSMCs, respectively, $\Phi_{e}+\Phi_{c}+\Phi_{\mathrm{VSMC}}=1$, and $\Phi_{e}\geq 0$, $\Phi_{c}\geq 0$ and $\Phi_{\mathrm{VSMC}}\geq 0$.

Elastin was modelled as a neo‐Hookean material:

(4) $$\Psi_{e}=\;\mu \left(I_{1,e}-3\right),$$

where μ is a stiffness‐like parameter, and $I_{1,e}$ is the first invariant of elastin's right Cauchy–Green tensor $C_{e}=F^{T}G_{e}^{T}G_{e}F$. $G_{e}$ is elastin's deposition stretch matrix with respect to $\kappa_{r}$:

(5) $$\begin{array}{c} \;G_{e}=\mathrm{diag}\left[\frac{1}{\lambda_{\theta ,e}\lambda_{z,e}},\;\lambda_{\theta ,e},\lambda_{z,e}\right] \end{array},$$

where $\lambda_{\theta ,e}$ and $\lambda_{z,e}$ are elastin's circumferential and axial deposition stretches, respectively.

Collagen was modelled as the superimposed contribution of four families of fibres:

(6) $$\Psi_{c}=\sum_{i=1}^{4}\frac{k_{1}^{i}}{4k_{2}^{i}}\left[e^{k_{2}^{i}{\left(I_{4,c}^{i}-1\right)}^{2}}-1\right],$$

where $k_{1}^{i}$, $k_{2}^{i}$, and $I_{4,c}^{i}$ are a stiffness‐like parameter, a non‐linearity parameter and the fourth invariant of the right Cauchy–Green tensor of the *i‐*th family of collagen fibres. The right Cauchy–Green tensor of the collagen fibre family i is $C_{c}^{i}=F_{1}^{T}{(G_{c}^{i})}^{T}G_{c}^{i}F_{1}$, with

(7) $$\begin{array}{c} \;C_{c}^{i}=\text{diag}\left[\frac{1}{\lambda_{c}^{2}\sin\alpha^{i}\cos\alpha^{i}},\lambda_{c}\sin\alpha^{i},\lambda_{c}\cos\alpha^{i}\right] \end{array},$$

where $\lambda_{c}$ is collagen deposition stretch in the fibre direction and $\alpha^{i}$ defines the orientation of the fibre family with respect to the vessel's axial direction. $\alpha^{1}=0^{\circ }$, $\alpha^{2}=90^{\circ }$, and $\alpha^{3,4}=\pm \alpha$ denote axially, circumferentially, and diagonally oriented fibre families, respectively.

Finally, we used Zulliger's formulation (2004) to describe the active contribution of VSMCs:

(8) $$\Psi_{\mathrm{VSMC}}=S_{1}\;S_{2}T_{\mathrm{VSMC}}\left(\hat{\lambda_{\theta }}-\ln\hat{\lambda_{\theta }}-1\right),$$

where $0\leq S_{1}\leq 1$ defines the level of VSMC contraction, $S_{2}$ controls the deformation range in which VSMCs are capable of generating force, $T_{\mathrm{VSMC}}$ is a stress‐like parameter, $\hat{\lambda_{\theta }}=\lambda_{\mathrm{VSMC}}\lambda_{\theta }$, and $\lambda_{\mathrm{VSMC}}$ is the circumferential deposition stretch of VSMCs. This model describes VSMC's contractile behaviour on the ascending limb of their parabolic force–length relationship, as VSMCs remain within this operating range in *in vivo‐*like conditions (Giudici et al., 2024). Notably, we assume VSMCs’ contribution to be only in the circumferential direction. Furthermore, we assume $S_{2}=1$ for $\hat{\lambda_{\theta }}\geq 1$ and $S_{2}=0$ otherwise, so that the active contribution of VSMCs is always positive (i.e., no contractile force generated under compression). Also, we do not set a deformation upper boundary for VSMC's ability to generate force, under the assumption that this upper limit is probably not reached *in vivo* at resting pressures.

### *In vivo* biomechanical data

The proposed biomechanical modelling framework was used to model the data of the study (NCT03594799) of Boutouyrie et al. (2022), who investigated the impact of 60 days of HDBR on arterial function. In their study, twenty healthy male participants spent 60 days on a bed tilted 6° head‐down to simulate a microgravity environment. Haemodynamic data, including local arterial waveforms of different arterial beds and carotid‐femoral pulse wave velocity, were acquired at five time points during the study: at baseline (B02), at day 29 and 52 of HDBR (H29 and H52, respectively), and at days 12 and 30 of the recovery period (i.e., resumed normal life, R12 and R30) (Fig. 1). In the present study, we focused on the local arterial waveforms acquired at the common carotid, common femoral and popliteal arteries. These consist of tonometry acquisitions of local pressure, ultrasound acquisitions of local diameter distension, and ultrasound measurements of the artery's intima‐media thickness (IMT). Because tonometry data were not available at the popliteal artery, these were replaced by femoral artery tonometry data, given the relative proximity between the two arterial beds and the similarity in arterial waveforms. A detailed description of the data acquisition is provided in Boutouyrie et al. (2022). All participants provided their informed consent to the experimental procedures, which were approved by the local ethics committee (CPP Sud‐Ouest et Outre‐Mer I, France, number ID RCB: 2016‐A00401–50) in accordance with the *Declaration of Helsinki*.

**Figure 1:**
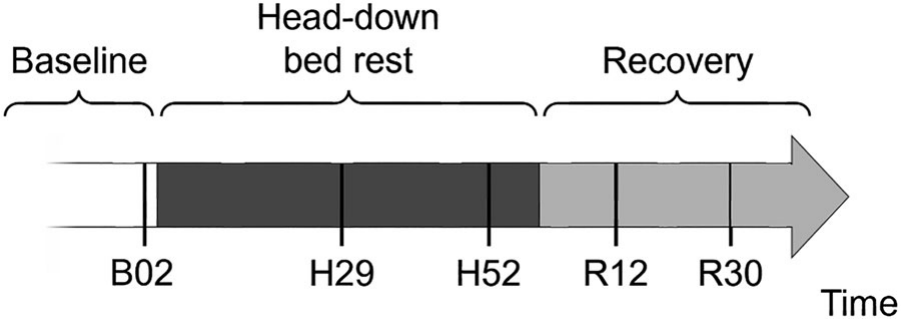
Schematic representation of the arterial waveform acquisition timeline in the 60‐day head‐down bed rest study by Boutouyrie et al. (2022) B02: baseline measurement performed 2 days prior to commencing the head‐down bed rest protocol; H29: 29^th^ day of head‐down bed rest; H52: 52^nd^ day of head‐down bed rest; R12: 12^th^ day of recovery; R30: 30^th^ day of recovery.

Both pressure and diameter distension data were pre‐processed to obtain representative waveforms of a single heartbeat for each arterial bed, for each study time point, and for each study participant (i.e., ensemble averaging). The foot of the waveforms was used as a fiducial point for the time alignment of different heartbeats within the same signal (i.e., pressure/diameter) before ensemble averaging. Then, we used the intersecting interpolant method to identify the systolic feet of both the pressure and diameter distension waveforms and took these as fiducial points for aligning the two signals, thereby obtaining a representative pressure–diameter loop over a cardiac cycle. Finally, taking the measured IMT as a measurement of wall thickness at end diastole ($h_{d}$), the wall thickness (h) at any point within the cardiac cycle was calculated from the incompressibility constraint assuming no change in the vessel axial length:

(9) $$\;h=\frac{-d+\sqrt{d^{2}+4\left(h_{d}^{2}+d_{d}h_{d}\right)}}{2}$$

where d is the vessel lumen diameter at a generic time point in the cardiac cycle, and $d_{d}$ is the vessel lumen diameter at end diastole. Overall, data processing yielded a pressure–diameter–thickness relationship at five time points (i.e., B02, H29, H53, R12 and R30) for each arterial bed (i.e., carotid, femoral and popliteal) of each study participant.

### Parameter estimation

The model described in the *Constitutive modelling framework* section involves *n* = 16 model parameters: four related to elastin (μ, $\lambda_{\theta ,e}$, $\lambda_{z,e}$, $\Phi_{e}$), eight related to collagen ($k_{1}^{1}$, $k_{2}^{1}$, $k_{1}^{2}$, $k_{2}^{2}$, $k_{1}^{3,4}$, $k_{2}^{3,4}$, α, $\lambda_{c}$) and four related to VSMCs ($S_{1}$, $T_{\mathrm{VSMC}}$, $\lambda_{\mathrm{VSMC}}$, $\Phi_{\mathrm{VSMC}}$). Note that the collagen volume fraction ($\Phi_{c}$) is known, given those of elastin and VSMCs ($\Phi_{c}=1-\Phi_{e}-\Phi_{\mathrm{VSMC}}$). Assuming that all collagen fibres have the same stiffness‐like parameter (i.e., $k_{1}^{1}=k_{1}^{2}=k_{1}^{3,4}=k_{1}$) reduces the number of parameters to *n* = 14. These fourteen parameters need to be estimated for each patient, for each arterial bed, and for each study time point (i.e., a total of *n* = 210 model parameters per participant).

Leveraging the longitudinal design of our study, we made assumptions to reduce the number of freely estimated model parameters. First, we postulated that the absolute content (i.e., volume) of elastin and VSMCs did not change between different study time points. Indeed, elastin is not produced in adulthood (Schmelzer & Duca, 2022), and the timeframe of our study can be considered too short (i.e., ∼3 months) to observe any significant ageing‐induced elastin degradation. Furthermore, because of their active nature, VSMCs are assumed to respond to remodelling primarily by altering their contraction rather than through proliferation. As such, changes in the wall volume between time points must be accounted for by the secretion or digestion of collagen fibres. Hence, the elastin and VSMC volume fractions at any time point can be defined as a function of their volume fraction at baseline:

(10) $$\begin{array}{c} \;\Phi_{i,t_{j}}=\Phi_{i,t_{1}}\;\frac{V_{a,t_{1}}}{V_{a,t_{j}}} \end{array},$$

where $\Phi_{i,t_{j}}$ is the volume fraction of the constituent i∈[e,VSMC] at time point $t_{j}$, and $V_{a,t_{j}}$ is the wall volume for vessel a∈[carotid,femoral,popliteal] at $t_{j}$, which can be determined from the IMT and $d_{d}$ at time point $t_{j}$. Additionally, constituent volume fractions and stiffness‐like parameters multiply each other in eqn (3) (see also eqns 4, 6, and 8). This means that, for each vessel, there exists an infinite number of possible volume fraction–stiffness‐like parameter combinations yielding the same fit of the measured data. For this reason, we imposed $\Phi_{e,t_{1}}=0.20$ at all locations based on literature data and only fitted $\Phi_{\mathrm{VSMC},t_{1}}$ to the measured data (with $\Phi_{e,t_{j}}$ and $\Phi_{\mathrm{VSMC},t_{j}}$ following from eqn (10), and $\Phi_{c,t_{j}}$ following from $\Phi_{e,t_{j}}+\Phi_{c,t_{j}}+\Phi_{\mathrm{VSMC},t_{j}}=1$). In the Appendix, Fig. A1 illustrates how this strategy ensured the convexity of the cost function in the $\Phi_{\mathrm{VSMC},t_{j}}$–$T_{\mathrm{VSMC}}$ plane. These assumptions reduce the total number of unknown parameters to *n* = 183.

Second, we conveniently defined $\kappa_{r}$ as the vessel configuration at mean blood pressure at baseline and defined all model parameters at subsequent time points with respect to this baseline configuration (note, however, that the wall thickness in $\kappa_{r}$ changed at different time points to reflect measured changes in IMT throughout the study). Notably, the choice of a time independent reference configuration $\kappa_{r}$ challenges the usual definition of ‘deposition stretches’, as seen, for example, in growth and remodelling frameworks (Latorre & Humphrey, 2018). In our work, the constituents’ deposition stretches at each time point should be interpreted as the deformation that the individual constituents would experience if the vessel returned to the baseline reference configuration. Because we assume the elastin matrix remains unchanged throughout the studied timeframe, $G_{e}$ becomes constant in each vessel. Furthermore, in accordance with our previous work (Giudici, van der Laan, et al., 2023), we assume the elastin matrix to be stress‐free in the vessel's unloaded configuration, which, however, is unknown from *in vivo* data. Therefore, we take literature axial stretch values measured upon *ex vivo* excision in human donors (Learoyd & Taylor, 1966): $\lambda_{z,e}=$ 1.22 at the carotid and 1.32 at the popliteal and femoral arteries. Furthermore, by assuming an equibiaxial deformation of elastin at physiological pressures, equal values are also imposed for $\lambda_{\theta ,e}$. These assumptions further reduce the number of unknown parameters to *n* = 153.

Third, we assume the intrinsic stiffness of all constituents (i.e., μ, $k_{1}$, and $T_{\mathrm{VSMC}}$) to be unaffected by wall remodelling and to be equal at all arterial locations. Similarly, collagen's non‐linearity parameters ($k_{2}^{1}$, $k_{2}^{2}$, and $k_{2}^{3,4}$), which describe the gradual recruitment of collagen fibres, are assumed not to change with time, albeit potentially differing between arterial beds. These assumptions further reduce the dimensionality of the problem to *n* = 75 parameters.

Finally, $S_{1}$ and $T_{\mathrm{VSMC}}$ multiply each other in eqn (8), which means that there exists an infinite number of possible combinations yielding the same modelled VSMC behaviour. To address this issue, we assumed that all three arterial beds presented the same level of vascular tone at baseline ($S_{1,t_{1}}=0.30$) (Latorre & Humphrey, 2018) and determined all other contraction levels ($0\leq S_{1,t_{j}}\leq 1$) with respect to this baseline contraction.

After these considerations, the final number of freely estimated parameters reduces to *n* = 72 for each participant (Table 1), which were simultaneously fitted to the *in vivo* biomechanical data, consisting of fifteen measured pressure–diameter relationships (i.e., one per time point at each arterial location). Additionally, at their *in vivo* length, arteries exhibit a relatively constant reduced axial force (F) over a wide range of physiological pressures (van Loon et al., 1977). F is computed as:

(11) $$\begin{array}{c} F=\pi \left(2\sigma_{\mathrm{zz}}-\sigma_{\theta \theta }-\sigma_{\mathrm{rr}}\right)r_{m}h \end{array},$$

where $\sigma_{ii}$ is the Cauchy stress in the direction ii. As such, constancy of F in the range 50–180 mmHg was also involved in the parameter estimation. Finally, with our model, we aimed to capture well changes in stiffness between different time points (i.e., the slope of the pressure–diameter relationship). As such, the cost function also included local PWV, defined as:

(12) $$\mathrm{PWV}\;=d_{d}\;\sqrt{\frac{\mathrm{SBP}-\mathrm{DBP}}{\rho \left(d_{s}^{2}-d_{d}^{2}\right)}}$$

where SBP and DBP are the systolic and diastolic blood pressure, $d_{s}$ is the lumen diameter at peak systole, and ρ=1050 kg m^−3^ is the blood mass density. Notably, inclusion of PWV in the cost function ensures that in case two parameter solutions yield similar matching of the measured pressure–diameter relationship, the one with the closest slope is considered the best fit of the data.

**Table 1:** List of the model parameters and details on their estimation.

Constituent	Parameter	Differs between arteries?	Differs between time points?	Estimated from?	*N*	Fit range
Elastin	μ [kPa]	No	No	1^st^ optimisation	1	[1−300]
	$\lambda_{\theta ,e}$ [–]	Yes	No	Literature (Learoyd & Taylor, 1966)	0	
	$\lambda_{z,e}$ [–]	Yes	No	Literature (Learoyd & Taylor, 1966)	0	
	$\Phi_{e}$ [–]	No	No	Literature (Cox, 1978)	0	
Collagen	$k_{1}$ [kPa]	No	No	1^st^ optimisation	1	[1−300]
	$k_{2}^{1}$ [–]	Yes	No	2^nd^ optimisation	3	(0−120]
	$k_{2}^{2}$ [–]	Yes	No	2^nd^ optimisation	3	(0−120]
	$k_{2}^{3,4}$ [–]	Yes	No	2^nd^ optimisation	3	(0−120]
	α [°]	Yes	Yes	2^nd^ optimisation	15	[0−90]
	$\lambda_{c}$ [–]	Yes	Yes	2^nd^ optimisation	15	[0.7−1.5]
VSMC	$T_{\mathrm{VSMC}}$ [MPa]	No	No	1^st^ optimisation	1	(0−1.2]
	$S_{1}$ [–]	Yes	Yes	2^nd^ optimisation	12^*^	[0−1]
	$\lambda_{\mathrm{VSMC}}$ [–]	Yes	Yes	2^nd^ optimisation	15	[0.7−1.7]
	$\Phi_{\mathrm{VSMC}}$ [–]	Yes	Yes	2^nd^ optimisation	3^#^	[0−0.8]

*N* indicates the number of times a given parameter must be estimated for each model fitting (i.e. also accounting for the three different arterial beds and five different time points). Hence, *N = *1 for a parameter that does not change between arteries and is constant with time, *N = *3 for a parameter that changes between arteries but not with time, *N = *3 × 5 = 15 for a parameter that changes between arteries and with time, and *N = *0 when a parameter is taken from literature.

^*^ As described in the Parameter estimation section, $S_{1}$ is assumed to equal 0.30 at baseline for all three arteries.

^#^ Because the time dependency of $\Phi_{\mathrm{VSMC}}$ follows from changes in vessel volume (eqn 10), only $\Phi_{\mathrm{VSMC}}$ at baseline must be fitted to *in vivo* data.

Model parameters are estimated through two optimisations. The first optimisation is performed on all three arteries simultaneously (72 model parameters) and is used to estimate the three location independent model parameters (μ, $k_{1}$ and $T_{\mathrm{VSMC}}$). The second estimation is performed individually on the three different arterial beds (i.e. repeated three times) and is used to estimate the remaining 23 parameters at each location.

μ: elastin's stiffness‐like parameter; $\lambda_{\theta ,e}$: elastin's circumferential deposition stretch; $\lambda_{z,e}$: elastin's axial deposition stretch; $\Phi_{e}$: elastin volume fraction; $k_{1}$: collagen's stiffness‐like parameter; $k_{2}^{1}$: collagen's non‐linearity parameter for axially oriented fibres; $k_{2}^{2}$: collagen's non‐linearity parameter for circumferentially oriented fibres; $k_{2}^{3,4}$: collagen's non‐linearity parameter for diagonally oriented fibres; α: orientation angle of diagonally oriented fibres; $\lambda_{c}$: collagen's deposition stretch in the fibre direction; $T_{\mathrm{VSMC}}$: vascular smooth muscle cells (VSMCs)’ stress‐like parameter; $\lambda_{\mathrm{VSMC}}$: VSMCs’ deposition stretch; $\Phi_{\mathrm{VSMC}}$: VSMCs’ volume fraction. Note that the collagen volume fraction can be determined as $\Phi_{c}=1-\Phi_{e}-\Phi_{\mathrm{VSMC}}$.

The cost function was, hence, defined as:

(13) $$\begin{array}{c} \Pi =\sum_{a=1}^{3}\left[\sum_{t_{j}=t_{1}}^{t_{5}}w^{P}\Pi_{a,t_{j}}^{P}+w^{F}\Pi_{a,t_{j}}^{F}+w^{\mathrm{PWV}}\Pi_{a,t_{j}}^{\mathrm{PWV}}\right] \end{array},$$

where subscript *a* and $t_{j}$ indicate an artery and a time point, and $w^{l}$ and $\Pi_{a,t_{j}}^{l}$ with l∈{P,F,PWV} are weights and error terms for the pressure, reduced axial force, and PWV terms of the cost function. $\Pi_{j,k}^{P}$ is defined as:

(14) $$\Pi_{a,t_{j}}^{P}=\sum_{n=1}^{N}{\left(\frac{P_{\mathrm{mod},n}-P_{\exp,n}}{P_{\exp,n}}\right)}^{2},$$

where $P_{\mathrm{mod},n}$ and $P_{\exp,n}$ are the measured and modelled pressure for the *n*‐th datapoint in the pressure–diameter curve, and N is the total number of datapoints. Given eqns ((1), (2), (3), (4), (5), (6), (7), (8)), $P_{\mathrm{mod},n}$ can be determined as:

(15) $$\begin{array}{c} \;P_{\mathrm{mod}}=\left(\sigma_{\theta \theta }-\sigma_{\mathrm{rr}}\right)\frac{h}{r_{m}} \end{array},$$

with

(16) $$\sigma_{\mathrm{ii}}=\;-p+2\frac{\partial \Psi }{\partial \lambda_{i}^{2}}\lambda_{i}^{2}.$$

*p* is a Lagrange multiplier enforcing incompressibility that can be determined from the radial stress equation: $-p+2\frac{\partial \Psi }{\partial \lambda_{r}^{2}}\lambda_{r}^{2}=-\frac{P}{2}$.

$\Pi_{a,t_{j}}^{F}$ was defined as:

(17) $$\;\Pi_{a,t_{j}}^{F}=\sum_{n=1}^{N}{\left(\frac{F_{\mathrm{mod},n}-F_{a,t_{j}}^{\mathrm{target}}}{F_{a,t_{j}}^{\mathrm{target}}}\right)}^{2},$$

where the modelled force $F_{\mathrm{mod},j}$ is given by eqns (11) and (16), and:

(18) $$F_{a,t_{j}}^{\mathrm{target}}=F_{a,t_{1}}^{\mathrm{target}}\frac{V_{a,t_{j}}}{V_{a,t_{1}}}.$$

The target force at the first time point was calculated from eqn (11) evaluated at end diastole, taking $\sigma_{rr}=-\frac{P}{2}$ and assuming $\sigma_{zz}≅\sigma_{\theta \theta }=P\frac{d_{d}}{2h_{d}}$ as a result of biomechanical homeostasis (Humphrey, 2008). Note that $F_{\mathrm{mod},n}$ was evaluated at N equally‐spaced circumferential stretch datapoints corresponding to the pressure range 50–180 mmHg.

$\Pi_{a,t_{j}}^{\mathrm{PWV}}$ was determined as:

(19) $$\Pi_{a,t_{j}}^{\mathrm{PWV}}={\left(\frac{\mathrm{PW}V_{\mathrm{mod},t_{j}}-\mathrm{PW}V_{\exp,t_{j}}}{\mathrm{PW}V_{\exp,t_{j}}}\right)}^{2},$$

where $\mathrm{PW}V_{\mathrm{mod},t_{j}}$ and $\mathrm{PW}V_{\exp,t_{j}}$ are determined by applying eqn (12) to the modelled and measured pressure–diameter relationship at time $t_{j}$. Lastly, we set $w^{P}=1$, $w^{F}=1/10$ and $w^{\mathrm{PWV}}=N/10$ to achieve a 10:1:1 weighting between the pressure, force and PWV terms in eqn (13).

The model parameters were estimated using the MATLAB functions *lsqnonlin* and *multistart* (version 2025a, The MathWorks, Inc., Natick, MA, USA) to repeat the least square optimisation starting from *n* = 100 initial guesses of model parameters. Because of the high number of model parameters and the probable existence of several local minima in the parameter space, we ran two subsequent optimisations. For each participant, the first optimisation was conducted on the entire dataset (i.e., carotid, femoral, and popliteal data). From this first optimisation, we saved the values of the model parameters that are shared between arterial locations, that is, the stiffness‐like parameters μ, $k_{1}$, and $T_{\mathrm{VSMC}}$. Then, the second optimisation was run separately for each artery, also constraining μ, $k_{1}$ and $T_{\mathrm{VSMC}}$ to values determined with the first optimisation. Our preliminary analysis indicated that *n *= 100 initial guesses of model parameters allowed for a consistent and reliable identification of the cost function global minimum.

### Estimation of biomechanical variables

The constitutive model and estimated model parameters were used to calculate biomechanical variables of interest at the reference pressure of 100 mmHg and for two scenarios: (i) *in vivo* contractile state (passive + active; i.e., using the $S_{1}$ value determined during the parameter estimation) and (ii) passive mechanics (i.e., imposing $S_{1}=0$ at all time points for all vessels). The latter scenario allowed for isolating the effect of HDBR‐induced wall remodelling on the extracellular matrix (ECM) biomechanics. The circumferential and axial components of the Cauchy stress tensor were calculated using eqn (16). The vessel material stiffnesses was determined according to the small‐on‐large framework (Baek et al., 2007). The vessel (circumferential) structural stiffness was determined in terms of PWV, using eqn (12) over the pressure range 80–120 mmHg. The individual constituent circumferential load bearings were determined as:

(20) $$\begin{array}{c} \;\Gamma^{i}=\frac{\sigma_{\theta \theta }^{i}}{\sigma_{\theta \theta }}\;\cdot 100\% \end{array},$$

where i∈{e,c,VSMC} and $\sigma_{\theta \theta }^{i}$ is the circumferential Cauchy stress borne by constituent *i*, which can be calculated by applying eqn (16) on $\Phi_{i}\Psi_{i}$.

### Statistical analysis

Differences in biomechanical variables between arteries at the five time points were statistically evaluated through mixed‐effect models considering repeated measures along two independent variables: the three arterial beds and the five time points. This was followed by Tukey's *post hoc* tests for multiple comparisons between arteries at a given time point and between time points for a given artery. Statistical analyses were run in Prism, version 10 (GraphPad Software Inc., San Diego, CA, USA). *p *< 0.05 was considered statistically significant.

## Results

### Modelled pressure–diameter relationships

The proposed modelling framework captured well the behaviour of the carotid (*R* ^2^ = 0.97 ± 0.01), femoral (*R* ^2^ = 0.84 ± 0.10) and popliteal arteries (*R* ^2^ = 0.88 ± 0.06). Two participants had missing data for at least one artery at one of the time points (participants D1 and F2). In these cases, the model was fitted to the remaining data. Figure 2 presents average modelled pressure–diameter relationships at the three arterial beds, at the five different time points considered in this study, and in *in vivo* (Fig. 2*A,C* and *E*) and passive (Fig. 2*B,D* and *F*) contractile conditions. Model parameters of each artery at all time points are reported in the Appendix (Tables A1, A2, A3, A4, A5).

**Figure 2:**
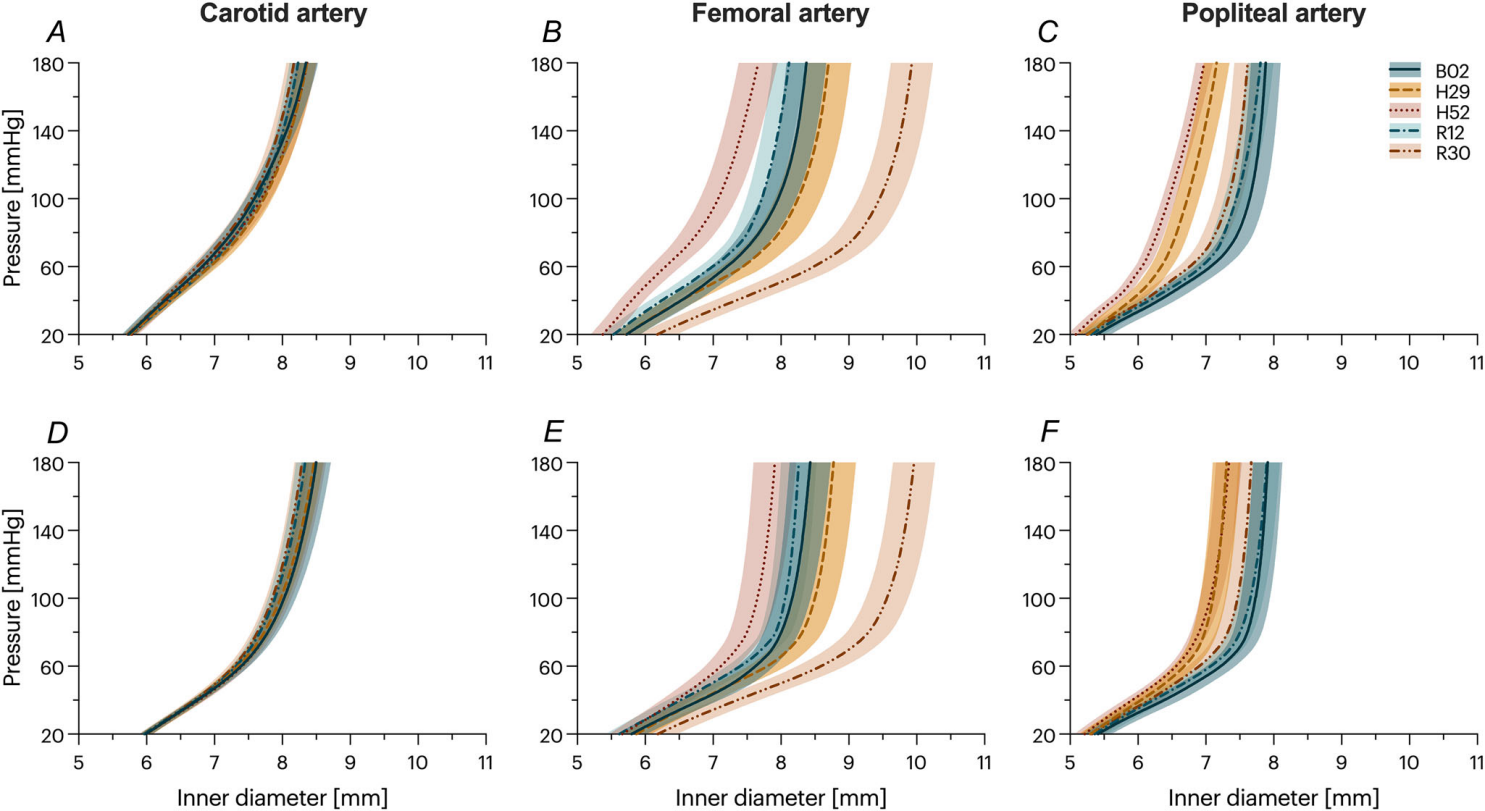
Average modelled pressure–diameter relationship of the carotid (*A* and *D*), femoral (*B* and *E*) and popliteal (*C* and *F*) arteries at the five time points of the study and in the *in vivo* (active + passive) (*A*–*C*) and passive contractile states (*D*–*F*) Lines indicate the average pressure–diameter relationship (in the diameter direction), and shaded areas indicate ±1 standard error around this average. Example of fitted pressure–diameter relationships are provided in the Appendix (Fig. A2). B02: baseline; H29: 29^th^ day of head‐down bed rest; H52: 52^nd^ day of head‐down bed rest; R12: 12^th^ day of recovery; R30: 30^th^ day of recovery.

The behaviour of the carotid artery remained remarkably similar throughout the study, with only a marginal increase in pressure–diameter non‐linearity at the three intermediate time points (H29, H52, and R12 vs. B02 and R30) (Fig. 2*A*). Conversely, both femoral and popliteal arteries underwent considerable changes in biomechanical behaviour in response to HDBR, also exhibiting some differences between the two sites. The femoral artery increased in diameter in the first 29 days of HDBR. Then, its diameter decreased between days 29 and 52 of HDBR, which was also accompanied by a decrease in slope and non‐linearity of the pressure–diameter relationship (Fig. 2*C*). During the recovery period, the femoral artery underwent a large dilatation (surpassing its baseline diameter at R30), while recovering the non‐linearity in pressure–diameter relationship. The popliteal artery followed a similar trend of reduction in diameter during HDBR, followed by a dilatation during the recovery period. However, pressure–diameter relationships at B02 and R30 were much more similar (in both shape and diameter range) compared to those of the femoral artery (Fig. 2*B*).

Computationally removing VSMCs’ contractile function attenuated differences in pressure–diameter relationship between time points, particularly for the femoral and popliteal artery (Fig. 2*D* and *F*).

### Biomechanical variables in the *in vivo* (passive + active) contractile condition

Figure 3 presents the calculated biomechanical variables for the carotid, femoral, and popliteal arteries, respectively. The main effects and *post hoc* results of the mixed model are reported in Fig. 3 and Table 2. Despite the observed changes in arterial diameter between time points (especially at the femoral and popliteal arteries, Fig. 2*C* and *D*), average circumferential wall stresses remained relatively uniform across arteries and constant throughout the duration of the study (Fig. 2*A*). Only, at the popliteal artery, the wall stress dropped significantly during HDBR, becoming significantly lower than the circumferential stresses at the carotid and femoral arteries.

**Figure 3:**
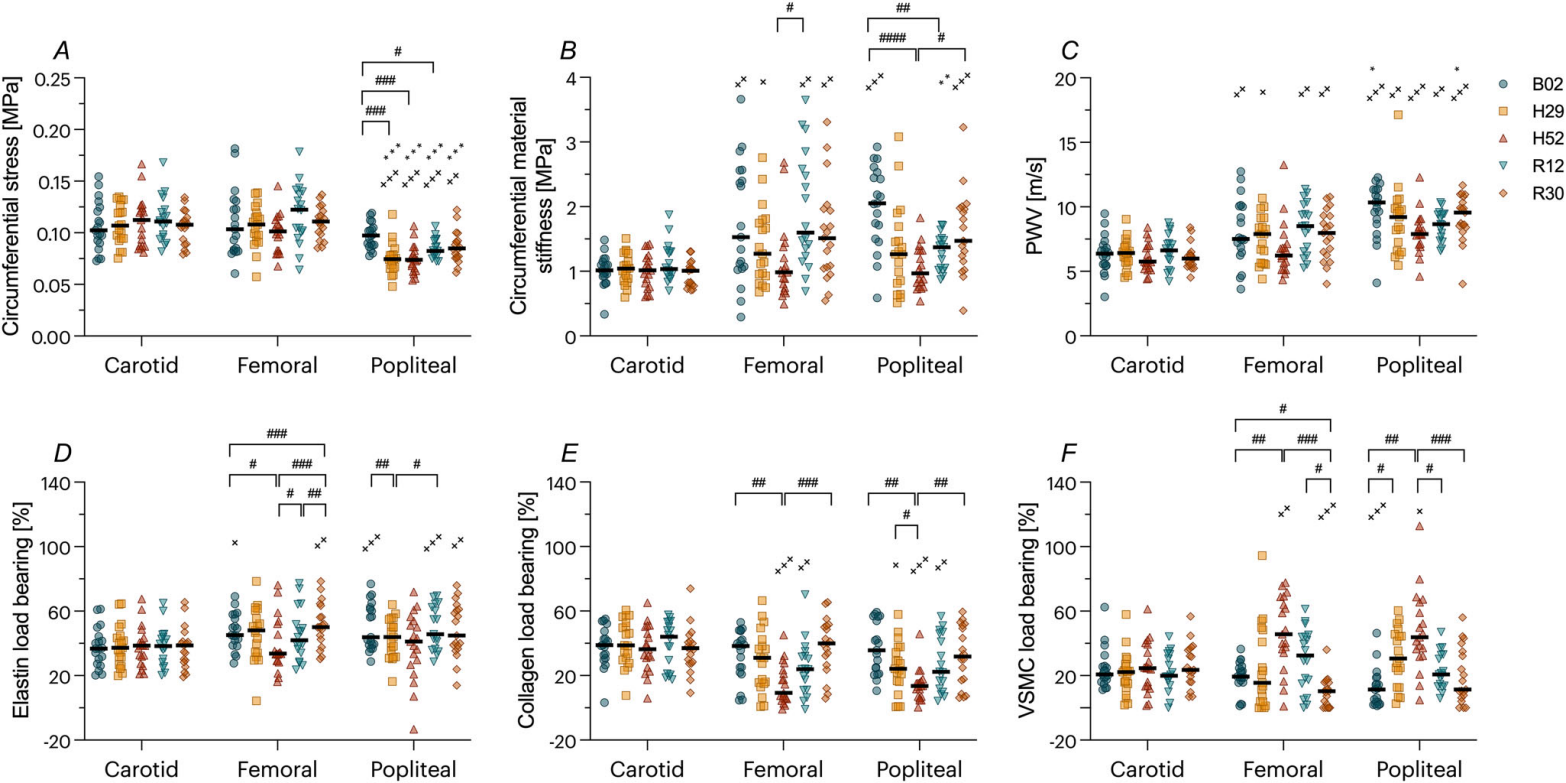
Changes in biomechanical variables between the five study time points (B02: baseline, H29 and H52: 29^th^ and 52^nd^ day of head down bed rest, respectively, and R12 and R30: 12^th^ and 30^th^ days of recovery) at the three arterial sites (carotid, femoral, and popliteal artery) in *in vivo* conditions *A*, circumferential stress. *B*, circumferential stiffness. *C*, local pulse wave velocity. *D*, elastin load bearing. *E*, collagen load bearing. *F*, vascular smooth muscle cells (VSMCs) load bearing. Black horizontal lines indicate medians. #*p *< 0.05, ##*p *< 0.01 and ###*p *< 0.001 between time points of variables of the same artery, respectively. Between artery comparisons at a given time point. +*p *< 0.05, ++*p *< 0.01 and +++*p *< 0.001 with respect to the carotid artery. *p < 0.05, **p < 0.01 and ***p < 0.001 with respect to the femoral artery.

**Table 2:** Results of two‐way repeated measure ANOVA with artery (i.e., carotid, femoral and popliteal artery) and time (i.e., the study timepoint) as independent variables.

	Artery	Time	Artery × time
***In vivo* (passive + active)**			
Circumferential stress	**<0.001**	**0.016**	**0.015**
Circumferential stiffness	**<0.001**	**<0.001**	**0.004**
Pulse wave velocity	**<0.001**	**0.033**	0.191
Elastin load bearing	**0.027**	**<0.001**	**0.014**
Collagen load bearing	**0.001**	**<0.001**	**0.001**
VSMC load bearing	0.568	**<0.001**	**<0.001**
**Passive**			
Circ. stress	**<0.001**	0.225	0.336
Circ. stiffness	**<0.001**	0.244	0.107
Pulse wave velocity	**<0.001**	0.336	0.114
Elastin load bearing	**0.016**	**0.002**	**0.041**
Collagen load bearing	**0.015**	**0.002**	**0.042**

Statistically significant *post hoc* comparisons are shown in Figs 2 and 3. Boldfacing was used to highlight the statistically significant terms.

Conversely, wall stiffness dropped during HDBR at both the femoral and popliteal arteries and then increased towards baseline values during the recovery period (Fig. 3*B*, statistically significant only at the popliteal artery), whereas it did not change at the carotid artery. While there was a clear central‐to‐peripheral material stiffness gradient at baseline (carotid: 1.01 ± 0.24 MPa, femoral: 1.75 ± 0.91 MPa, and popliteal: 2.00 ± 0.62 MPa) and at day 30 of the recovery period (0.95 ± 0.19, 1.59 ± 0.73, and 1.60 ± 0.62, respectively), the three arteries showed similar stiffnesses at day 52 of HDBR (1.00 ± 0.26, 1.14 ± 0.58, and 1.05 ± 0.32, respectively).

At baseline, the central‐to‐peripheral stiffness gradient was also visible in terms of PWV (Fig. 3*C*). As for material stiffness, PWV differences between carotid and femoral arteries dropped during HDBR. However, this change was less accentuated at the popliteal artery, where PWV was significantly higher than both carotid and femoral PWVs at baseline and remained higher than carotid PWV at day 52 of HDBR. Once more, the central‐to‐peripheral stiffness gradient at the end of the study was similar to that at baseline.

Our constitutive modelling framework also provided insights into the relative contribution to the artery biomechanics of major mechanically relevant wall constituents (elastin, collagen, and VSMCs) at the three arterial locations (Fig. 3*D‐F*). Constituent load bearing was unaffected by the study protocol at the carotid artery. Conversely, VSMC load bearing significantly increased and decreased during HDBR and recovery, respectively, at both the femoral and popliteal arteries (Fig. 3*F*), as also evidenced by the contraction parameter $S_{1}$ (see Appendix Tables A4 and A5). Increased VSMC contraction predominantly unloaded collagen fibres (Fig. 3*E*), although elastin load bearing was also significantly reduced, especially at the femoral artery (Fig. 3*D*).

### Biomechanical variables in passive contractile condition

Figure 4 presents the calculated biomechanical variables for the carotid, femoral, and popliteal arteries, respectively, in passive conditions. Main effects and *post hoc* results of the mixed‐effect analysis are reported in Fig. 4 and Table 2. Overall, removing VSMC contraction led to higher circumferential wall stress, circumferential material stiffness, and PWV (Fig. 3*A‐C* vs. 4*A*–*C*). The circumferential wall stress in passive conditions followed a similar trend to that observed in the *in vivo* contractile state (passive + active), being significantly lower at the popliteal artery than at the carotid and femoral arteries (Fig. 4*A*). Only the stress at the popliteal artery decreased significantly during HDBR. Also in passive conditions, the central‐to‐peripheral stiffness gradient was present at baseline and attenuated during HDBR (in terms of both material stiffness and PWV). However, in contrast to the *in vivo* contractile state (passive + active), our model indicated that the passive central‐to‐peripheral stiffness gradient was not restored at the end of the recovery period, when the three arteries (especially, carotid and femoral) still exhibited relatively similar levels of passive stiffness and PWV.

**Figure 4:**
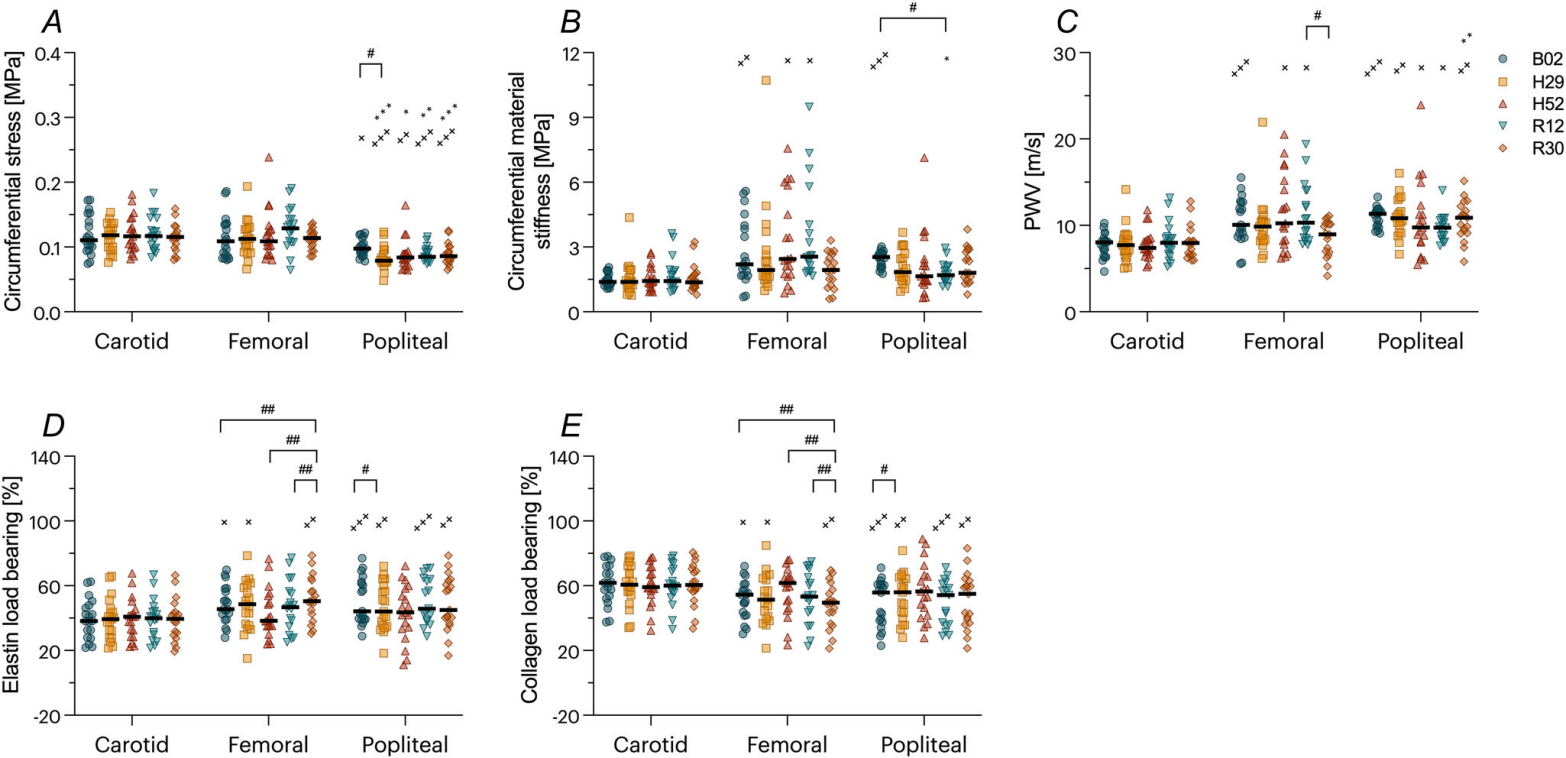
Changes in biomechanical variables between the five study time points (B02: baseline, H29 and H52: 29^th^ and 52^nd^ day of head down bed rest, respectively, and R12 and R30: 12^th^ and 30^th^ day of recovery) at the three arterial sites (carotid, femoral, and popliteal artery) in passive conditions *A*, circumferential stress. *B*, circumferential stiffness*. C*, local pulse wave velocity*. D*, elastin load bearing. *E*, collagen load bearing. Fully passive conditions were simulated by setting the contraction parameter $S_{1}=0$. Black horizontal lines indicate medians. #*p *< 0.05, ##*p *< 0.01 and ###*p *< 0.001 between time points of variables of the same artery, respectively. Between artery comparisons at a given time point. +*p *< 0.05, ++*p *< 0.01 and +++*p *< 0.001 with respect to the carotid artery. *p < 0.05, **p < 0.01 and ***p < 0.001 with respect to the femoral artery.

As in the *in vivo* contractile state, constituent load bearing in passive conditions did not change throughout the study at the carotid artery (Fig. 4*D*). Conversely, at the femoral and popliteal arteries, the load bearing marginally shifted from elastin to collagen during HDBR (by, on average, ∼5% and 6%, respectively) (Fig. 4*D* and *E*). Our model attributed this shift to collagen remodelling, with the collagen deposition stretch $\lambda_{c}$ increasing during HDBR compared to baseline at both the femoral and popliteal artery (see Appendix, Tables A4 and A5). Biomechanically, a higher $\lambda_{c}$ indicates earlier recruitment of collagen with vessel distension. During the recovery, the load shifted back to elastin, with femoral elastin load bearing being even significantly higher at R30 than B02. Unsurprisingly, $\lambda_{c}$ decreased towards or even below baseline values during the recovery period.

## Discussion

In the present study, we proposed a novel computational framework for the *in vivo* parameterisation of comprehensive arterial constitutive models incorporating the micromechanics of elastin, collagen, and VSMCs. This framework leverages longitudinal arterial waveform data acquired at different arterial sites to reduce the number of freely estimated model parameters, thereby enabling parameter estimation based on sparse, uniaxial *in vivo* biomechanical data, specifically non‐invasively measured pressure–diameter relationships. Application of this framework to data from the study by Boutouyrie et al. (2022) effectively disentangled the (micro)structural remodelling processes of central and peripheral arteries in response to altered haemodynamics during and after 60 days of HDBR, which is used to simulate microgravity environments on Earth. Our findings were as follows: (i) leg arteries (i.e., femoral and popliteal) undergo more intensive (micro)structural remodelling in response to HDBR than the carotid artery; (ii) despite large changes in vessel diameters (Fig. 2*B* and *C*), circumferential wall stresses remained relatively constant throughout the study duration as well as uniform across different arterial beds (except for a small, but significant drop at the popliteal artery); (iii) HDBR led to a reversible de‐stiffening of lower limb arteries, thereby reducing the central‐to‐peripheral stiffness gradient; and (iv) at a microstructural level, changes in arterial biomechanics in response to HDBR were attributable to two superimposed remodelling processes. On the one hand, a HDBR‐induced increase in the contraction of VSMCs shifted load bearing from the ECM (mainly collagen) to VSMCs. On the other hand, an alteration in the collagen fibre microstructural arrangement yielded an earlier recruitment of collagen fibres (i.e., at smaller diameter) during HDBR.

Similar to several other physiological processes, arterial wall remodelling aims to preserve a stable and optimal organ function, i.e., homeostasis (Humphrey, 2008). This process is enacted by cells (primarily endothelial cells, VSMCs, and fibroblasts) residing in the wall tissue, which can sense and respond to changes in their biomechanical environment. For example, endothelial cells sense the blood flow in the vessel lumen through cilia on their membranes. The sensed shear stress is known to be a crucial driver for both short‐ (i.e., vasoconstriction/vasodilatation) and long‐term (i.e., arterial remodelling) vascular adaptations (Humphrey, 2008; Kamiya & Togawa, 1980). Furthermore, VSMCs are recognised to play a pivotal role in sensing pressure‐induced wall stress, although the exact sensing mechanism is currently not fully understood. Through modulations of their phenotype (i.e., switching between contractile and synthetic behaviours), VSMCs can alter both arterial mechanics (i.e., contraction) and (micro)structure (i.e., wall thickness and composition) to maintain wall stress within physiological levels (Spronck et al., 2021; Vaishnav et al., 1990).

Two primary haemodynamic alterations probably drove the observed HDBR‐induced wall remodelling. First, changes in body position alter the hydrostatic pressure gradient acting in the arterial network. Whenever standing upright, foot arteries are exposed to a higher pressure (∼110–160 mmHg) than cerebral arteries (Petersen et al., 2013). Conversely, lying supine yields a more uniform pressure distribution across the body. During HDBR, the feet are located at a slightly higher height than the head (14–20 cm). This leads to a hydrostatic pressure unloading of leg arteries while maintaining a relatively physiological column of blood above the carotid artery. This shift would therefore be expected to trigger remodelling at the femoral and popliteal arteries to preserve wall stress. At first glance, our results do, indeed, suggest that HDBR‐induced remodelling preserved the circumferential wall stress within an optimal range both across different arterial beds and in response to altered haemodynamics (Fig. 3*A*). However, it is worth considering that we calculated circumferential stresses at a uniform pressure of 100 mmHg without accounting for expected/potential differences in average pressure exposures between different time points and arterial sites. This choice was dictated by the fact that an accurate estimation of actual changes in average pressure loading at each artery would require continuous monitoring of blood pressure and body position (especially at baseline and during recovery), which was unavailable in our data. This limitation prevents more accurate evaluations of wall stress changes throughout the study. Simulated femoral and popliteal circumferential stresses probably underestimated real stresses at baseline and during recovery, which would challenge the driving role of wall stress in arterial remodelling. Indeed, it is somewhat surprising to identify comparable baseline stresses at 100 mmHg across the three arteries, given that these arteries experience, on average, very different hydrostatic pressures (∼30 and 40 mmHg average daily mean pressure difference between the carotid and the femoral and popliteal arteries, respectively). Notably, this finding stems directly from the measured data (i.e., the interaction between the measured pressure, diameter and IMT; see eqn (15)) and is largely unaffected by modelling assumptions. Several factors may explain this unexpected finding. Our cohort manifested an increase in brachial diastolic blood pressure during HDBR, which may have compensated, at least in part, for the drop in blood pressure in the leg arteries (Boutouyrie et al., 2022). Furthermore, most literature evidence on arterial wall stress homeostasis focuses on arterial remodelling in response to increased haemodynamic loading (e.g., induced hypertension) (Spronck et al., 2021; Vaishnav et al., 1990), whereas VSMCs may be less susceptible to reduced wall stresses.

Blood flow is the second haemodynamic parameter that is altered, both globally and locally, during HDBR. Prolonged inactivity during HDBR leads to a decrease in cardiac output (Hoffmann et al., 2021). Because this drop is primarily related to the decreased oxygen demand of deambulation‐involved muscles, the relative blood flow distribution shifts towards arteries that supply vital organs, as their oxygen demand is largely unaffected by inactivity. Arterial diameter changes observed in our cohort reflect this flow redistribution. The diameter of the carotid artery did not change throughout the study, probably reflecting the unaltered demand for blood by the brain. Conversely, at both the femoral and popliteal arteries, average diameters (at 100 mmHg) decreased by 11% and 16% during HDBR, probably reflecting the decreased oxygen demands of leg muscles and consistent with previous observations in unilateral lower limb deconditioning (Bleeker, De Groot, Poelkens, et al., 2005). Indeed, in our cohort, blood flow velocities in the femoral and popliteal arteries decreased, on average, during HDBR (albeit not statistically significantly). Together with the concomitant significant drop in diameters, this is indicative of decreased local blood flows (Boutouyrie et al., 2022). Interestingly, although the popliteal artery returned to baseline diameters one month after recovery, at the end of the study, the femoral artery presented a 19% larger calibre compared to baseline (at 100 mmHg) (Fig, 2*B*). Although unaltered shear stresses at all arterial locations suggest that local changes in blood flow did drive those in diameter (Boutouyrie et al., 2022), the further dilatation of the femoral artery may have been induced by the intensive exercise regimen prescribed to participants after HDBR to stimulate their recovery. Indeed, previous work showed that (endurance) athletes present larger conduit arteries in body regions that are specific to the practised sport compared to untrained controls (Miyachi et al., 2001; Schmidt‐Trucksäss et al., 2000; Zeppilli et al., 1995). It is, hence, possible that thigh muscles were particularly challenged in the recovery phase, leading to a localised, heightened oxygen demand and, consequently, blood flow.

Our constitutive modelling framework provided insights into the microstructural remodelling processes that underlaid local changes in macroscopic arterial function. The increased VSMC load bearing during HDBR, both at the femoral and popliteal artery (Fig. 3*F*), suggests that observed changes in diameter during HDBR were partly caused by vasoconstriction. Nonetheless, upon imposing full vasodilatation (Fig. 2*E* and *F*), our model predicted a 6% and 9% contraction independent reduction in diameter at the femoral and popliteal arteries, respectively (at 100 mmHg). As such, our modelling work suggests that collagen is also remodelled during HDBR. In our model, the parameter $\lambda_{c}$ describes the pre‐deformation of collagen fibres in the reference configuration, which was defined as the configuration of the artery at mean blood pressure at baseline. For both femoral and popliteal arteries, $\lambda_{c}$ was, on average, higher during HDBR than at baseline (see Appendix Tables A4 and A5). Because the reference configuration was kept constant in our study, this change implies an earlier recruitment (i.e., at lower diameters) of collagen fibres during HDBR. This observation seems mechanobiologically plausible, as the deposition of new collagen fibres was probably optimised around the new working point resulting from a local adaptation to the new blood flow regime. The redistribution of loads between constituents was responsible for a reduction of the central‐to‐peripheral stiffness gradient during HDBR, which was observed in terms of both material and structural (i.e., local PWV) stiffness (Fig. 3*B* and *C*), as well as denoted by the increase in aortic PWV reported by Boutouyrie et al. (2022) (in terms of both regional carotid‐femoral PWV and magnetic resonance imaging‐based local PWV). Put simply, collagen is the stiffest constituent in the arterial wall, followed in stiffness by contracted VSMC, with elastin being the most compliant (Giudici & Spronck, 2022). As such, a vasoconstriction‐induced shift in load bearing from collagen to VSMCs yields a de‐stiffening of the artery wall, as observed at the femoral and popliteal arteries during HDBR (Fig. 3*B* and *C*). This effect appeared to dominate over that induced by the microstructural rearrangement of collagen fibres, which, in passive conditions, did not lead to stiffening despite a marginal shift in load bearing from elastin to collagen (Fig. 4*B*–*E*). A physiological central‐to‐peripheral stiffness gradient plays a pivotal role in dampening the transmission of pressure pulsatility to the peripheral circulation, thereby preventing damage to the microvasculature (Nichols et al., 2022). Although our data indicated that a physiological stiffness gradient could be recovered within 30 days after the termination of HDBR, they also suggest that an altered propagation of pressure waves may be a reason for concern during prolonged exposure to microgravity.

The sparsity of biomechanical data is the main barrier to applying complex constitutive models *in vivo*. In the present study, we proposed a novel framework that attempts to address this problem by (i) looking at changes in the biomechanical properties of arteries over time and (ii) observing these changes at different arterial beds simultaneously. Previous studies have employed constitutive models with six to fourteen model parameters to capture the biomechanics of arteries *in vivo*, with the number of parameters inherently reflecting the comprehensiveness and complexity of the proposed constitutive model (e.g., whether or not it accounts for VSMC contraction) (Åstrand et al., 2011; Gade et al., 2019, 2021; Heusinkveld et al., 2018; Masson et al., 2011; Spronck et al., 2015; Stålhand, 2009). However, it has been shown previously that parameter estimation from individual *in vivo* pressure–diameter curves can become problematic whenever models encompass more than four or five parameters (Heusinkveld et al., 2018; Parikh et al., 2024). Despite the comprehensiveness of the constitutive model proposed herein, which accounted for the mechanical contribution of elastin, collagen, and contracting VSMCs, we fitted ‘only’ 72 model parameters to 15 individual *in vivo* pressure–diameter curves, that is equivalent to <5 parameters per relationship (note, however, that most parameters were not determined from individual relationships; see Table 1). This parameter reduction was achieved through modelling assumptions, which primarily pertained to the evolution over time of the behaviour of individual constituents. Therefore, carefully considering these assumptions is crucial to evaluate our findings’ validity. First, we assumed that the absolute content of elastin and VSMCs would not change over time. Although this assumption is relatively straightforward for elastin, as a result of its long half‐life and lack of secretion in adulthood (Schmelzer & Duca, 2022), it may be reasonable to wonder whether VSMCs undergo atrophy during prolonged inactivity as observed in skeletal muscles. Although we did not find any relevant histological data in the literature, previous work has shown that the vasoactivity of the femoral artery (both endothelium‐dependent and endothelium independent) is preserved, if not increased during immobilisation‐induced unilateral lower limb deconditioning (Bleeker, De Groot, Poelkens, et al., 2005) and HDBR (Bleeker, De Groot, Rongen, et al., 2005). Indeed, it is plausible (as also suggested by the data presented herein) that prolonged muscular inactivity would lead to increased activity of VSMCs because of their necessary adaptations to haemodynamic changes, thereby supporting the validity of our modelling assumption. Furthermore, these assumptions on the constancy of the elastin and VSMC volume fractions should be revisited when applying our framework to longitudinal studies with longer observation times, for example, by employing exponential decay functions to describe elastin's degradation with ageing (Latorre & Humphrey, 2018). Second, we postulated that modifications in the biomechanical behaviour of individual constituents originated from changes in their microstructural arrangement (e.g., deposition stretches and fibre arrangement) and not from alterations in their intrinsic properties (e.g., stiffness), which were assumed constant over time and across different arteries. This assumption appears reasonable and also constitutes the basis of well‐established growth and remodelling formulations (Latorre & Humphrey, 2018).

### Limitations and future work

Wall thickness is a key parameter for the formulation of arterial biomechanical models. Here, as commonly done in *in vivo* parameter identification studies, we used IMT as a proxy of wall thickness because the latter cannot be reliably measured *in vivo*. Although it is possible that the adventitia underwent specific remodelling/adaptation in response to HDBR, its primary biomechanical role is to shield the artery against high pressure levels (Giudici, Khir, et al., 2021), which is distant from the haemodynamic loading conditions during HDBR. Indeed, in a previous study, we estimated that the adventitia of the distal thoracic aorta bears only one third of the total pressure load at physiological pressures and in the absence of VSMC contraction (Giudici, Spronck, et al., 2023). Because VSMCs are predominantly located in the medial layer, the significant contributions of VSMCs to arterial mechanics at all locations predicted by our current model would suggest a further shift of load bearing from the adventitia to the media. Therefore, although our IMT‐based estimation of the wall thickness is a source of uncertainty, we consider the resulting errors in our biomechanical analyses to be relatively small.

The proposed framework leverages longitudinal observation of changes in the biomechanics of arteries to facilitate the parameterisation of a complex constitutive model. Although this approach did provide insights into arterial remodelling processes in response to HDBR, this dataset would also be valuable to evaluate whether growth and remodelling laws allow for predicting changes in arterial (micro)structure and biomechanics given the measured changes in haemodynamics (Latorre & Humphrey, 2018). Furthermore, we characterised the effects of HDBR on the function of individual arteries, albeit using data from different arterial beds to aid model parametrisation. In future work, integrating these individual arterial models into, for example, one‐dimensional circulation models will allow for evaluating the consequences of HDBR at a more systemic level.

Finally, our analysis was limited to male participants because we used data collected in a previous study (Boutouyrie et al., 2022) and no similar study has been conducted on participants of both sexes. Although future work should evaluate whether and to what extent arterial remodelling in response to HDBR differs between the two sexes, we consider that the computational framework proposed herein would be applicable also to data from female participants.

## Conclusions

In the present study, we developed a novel computational framework that leverages longitudinal, *in vivo* haemodynamic measurements to achieve a detailed characterisation of arterial biomechanics and its evolution over time. We used this approach to provide novel insights into the remodelling processes that characterise the arterial response to altered haemodynamics during and after 60 days of HDBR. In agreement with previous work, our findings suggest that arterial remodelling aimed to preserve wall and shear stress homeostasis, which was achieved through a simultaneous increase in VSMC contraction and a microstructural rearrangement of collagen fibres. Both these HDBR‐induced constituent‐level adaptations appeared to be reversible within 30 days. Future work is warranted to ascertain its potential utility as a tool for monitoring the progression of vascular ageing in clinical settings.

## Additional information

## Competing interests

The authors declare that they have no competing interests.

## Author contributions

A.G., R.M.B., B.S. and P.B. were responsible for the conception and design of the work. K.B., U.G., H.K., E.M., C.L., R.M.B. and P.B. were responsible for acquisition of the data. A.G. was responsible for analysis of the data. A.G., R.M.B., B.S. and P.B. were responsible for interpretation of the data. A.G. was responsible for writing the original draft. A.G., K.B., U.G., H.K., E.M., C.L., T.D., R.M.B., B.S. and P.B. were responsible for reviewing and editing. All authors approved the final version of the manuscript submitted for publication and agree to be accountable for all aspects of the work in ensuring that question related to the accuracy or integrity of any part of the work are appropriately investigate and resolved. All persons designated as authors qualify for authorship, and all those who qualify for authorship are listed.

## Funding

This study was funded by a Short Term Scientific Mission grant of the European Union COST Action 18216 VascAgeNet (Network for Research in Vascular Ageing) to A.G. and by the European Union's Horizon Europe research and innovation programme (Grant No. 101136728, AG, BS, RMB and PB). The NCT03594799 study was funded by the French centre d'etudes spatiales (CNES).

## Supporting information


Peer Review History

## Figures and Tables

**Figure A1:**
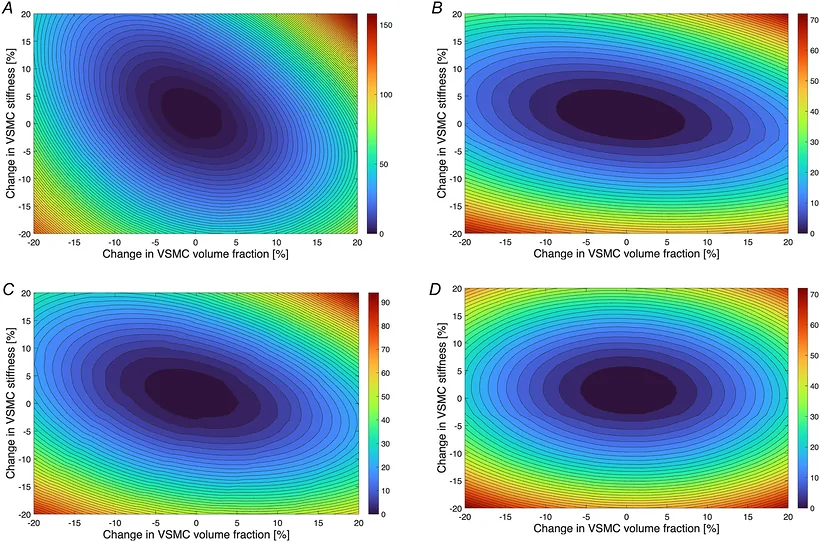
Identifiability analysis of the VSMC volume fraction and stiffness‐like parameter Percentage change of the cost function value (eqn 13) as a function of changes in VSMC volume fraction ($\Phi_{\mathrm{VSMC}}$) and stiffness‐like parameter ($T_{\mathrm{VSMC}}$). The change in VSMC volume fraction was applied uniformly on all arteries in (*A*) and individually at the carotid, femoral or popliteal artery alone in (*B*), (*C*) and (*D*), respectively. Colour bars indicate the percentage change in cost function value with respect to the best solution (i.e., when no change in VSMC stiffness and volume fraction was applied). $\Phi_{\mathrm{VSMC}}$ and $T_{\mathrm{VSMC}}$ multiply each other in eqn (3) (see also eqn (8)), potentially leading to inidentifiability issues (i.e., for all the $\Phi_{\mathrm{VSMC}}$–$T_{\mathrm{VSMC}}$ paris resulting in the same optimal product value). The parametrisation strategy proposed in this study, which exploits longitudinal acquisitions at three arterial beds, ensures the convexity of the cost function in the $\Phi_{\mathrm{VSMC}}$–$T_{\mathrm{VSMC}}$ plane and the existence of a unique solution. Unidentifiabililty would have resulted into a ‘valley‐shaped’ contour plot, with pairs of parameters in the valley equally minimising the cost function. The contour plot in (*A*) shows that this vally pattern partially persists, with the error gradient being steeper for a concomitant increase (top right) or decrease (bottom left) of the two parameters than for parameters changing in opposite directions (top left and bottom right).

**Figure A2:**
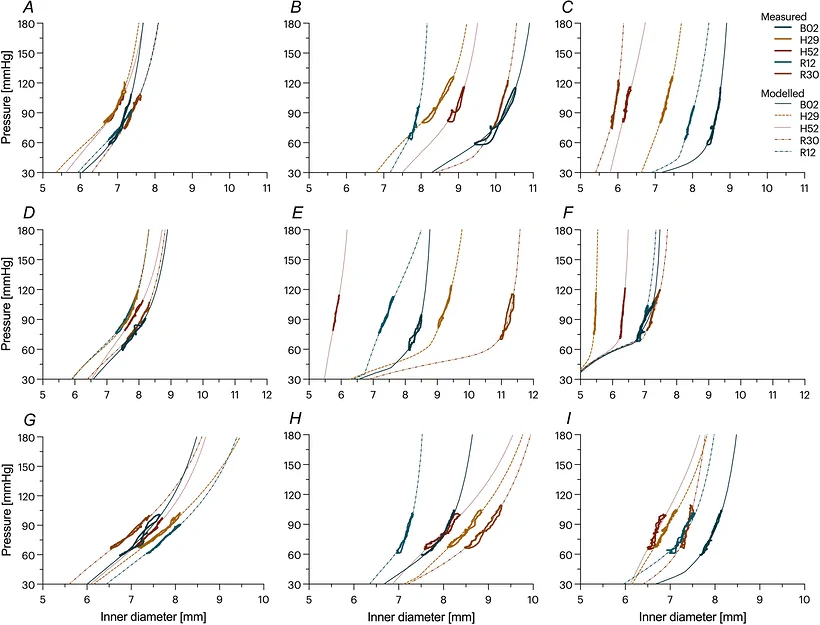
Example of fitted pressure–diameter relationships of the carotid (*A*, *D* and *G*), femoral (*B*, *E* and *H*) and popliteal (*C*, *F* and *I*) arteries of study participants (G2, B1 and H2, respectively) The study time points are: B02 = baseline, H29 = day 29 of head‐down bed rest (HDBR), H52 = day 52 of HDBR, R12 = day 12 of the recovery period and R30 = day 30 of the recovery period. Non‐continuities in the modelled pressure–diameter relationships (e.g., B02 and R30 in *H*) are a result of the assumption that VSMCs do not bear load under compression (see section on the Constitutive modelling framework).

## Data Availability

All data supporting this manuscript are available from the corresponding author upon reasonable request.

## Appendix

**Table A1:** Stiffness/stress‐like parameters of elastin, collagen and vascular smooth muscle cells for each participant.

Partecipant	A1	A2	B1	B2	C1	C2	D1	D2	E1	E2	F1	F2	G1	G2	H1	H2	I2	J1	J2	Mean	Av. behav.
μ [kPa]	176.0	273.6	218.6	150.1	103.3	123.7	173.6	133.7	178.5	149.2	280.0	139.7	185.7	138.8	176.6	134.2	206.0	155.2	222.1	**174.7**	**177.9**
$k_{1}$ [kPa]	41.2	22.6	13.2	17.2	77.6	68.8	58.2	108.0	56.2	39.6	66.9	49.4	46.7	33.0	37.7	101.7	65.9	61.2	19.3	**51.8**	**38.7**
$T_{\mathrm{VSMC}}$ [MPa]	0.99	0.86	1.11	1.13	1.13	1.20	1.10	1.06	1.08	0.75	0.69	0.48	1.20	1.20	0.98	0.71	1.19	1.03	1.14	**1.00**	**1.06**

Note that these parameters were assumed to be equal at all arterial beds and not to change over time. Average behaviour (Av. behav.) model parameters were obtained by fitting ensemble averaged pressure–diameter relationship across the entire cohort of participants with complete acquisition protocols (i.e., excluding D1 and F2). Boldfacing was use to highlight average parameter values in the cohort of 20 participants.

**Table A2:** Collagen non‐linearity parameters at the three arterial locations and for each participant.

Partecipant	A1	A2	B1	B2	C1	C2	D1	D2	E1	E2	F1	F2	G1	G2	H1	H2	I2	J1	J2	Mean	Av. behav.
**Carotid**																					
$k_{2}^{1}$ [‐]	2.3	2.6	4.0	5.9	0.2	0.2	2.3	3.5	25.3	0.3	7.5	2.4	2.5	3.7	3.1	0.1	5.6	7.3	0.0	**9.6**	**7.5**
$k_{2}^{2}$ [‐]	12.9	8.2	10.7	20.4	5.3	4.7	15.7	17.6	64.5	63.1	40.9	9.8	10.6	14.6	24.0	5.7	23.9	23.4	50.6	**4.1**	**2.8**
$k_{2}^{3,4}$ [‐]	0.0	0.0	7.6	2.0	6.2	0.0	9.2	14.5	0.3	71.5	0.0	2.9	13.5	11.8	31.8	5.8	5.9	0.0	0.0	**22.4**	**11.3**
**Femoral**																					
$k_{2}^{1}$ [‐]	4.9	0.0	7.3	0.0	0.0	0.0	0.0	6.6	26.7	0.0	90.0	0.0	0.0	0.8	15.3	8.0	68.0	0.0	90.0	**16.7**	**3.9**
$k_{2}^{2}$ [‐]	3.6	21.8	4.8	22.1	6.8	1.8	2.4	2.9	0.0	4.6	0.0	8.8	0.0	9.3	0.0	1.3	96.1	0.0	3.3	**10.0**	**2.8**
$k_{2}^{3,4}$ [‐]	120.0	73.0	48.4	89.9	25.6	52.8	89.1	108.4	34.3	89.9	77.7	34.6	89.5	24.3	37.0	30.6	67.2	86.2	78.6	**66.2**	**38.4**
**Popliteal**																					
$k_{2}^{1}$ [‐]	0.1	4.5	0.0	42.7	0.0	15.5	59.3	5.4	0.0	5.8	0.8	0.0	14.0	1.5	0.0	0.0	0.0	13.2	0.0	**8.6**	**1.1**
$k_{2}^{2}$ [‐]	0.0	21.6	26.0	0.0	21.7	0.0	0.0	5.7	18.3	6.7	23.4	29.5	18.9	20.5	19.9	8.3	42.6	29.0	0.0	**15.4**	**3.3**
$k_{2}^{3,4}$ [‐]	119.8	54.4	90.0	43.6	42.8	56.6	90.0	61.3	57.3	15.5	90.0	56.0	39.9	32.4	111.1	18.9	119.8	38.9	118.1	**66.1**	**36.7**

Note that these parameters were assumed not to change over time. Average behaviour (Av. behav.) model parameters were obtained by fitting ensemble averaged pressure–diameter relationship across the entire cohort of participants with complete acquisition protocols (i.e., excluding D1 and F2). Boldfacing was use to highlight average parameter values in the cohort of 20 participants.

**Table A3:** Time‐dependent model parameters of the carotid artery.

Partecipant	A1	A2	B1	B2	C1	C2	D1	D2	E1	E2	F1	F2	G1	G2	H1	H2	I2	J1	J2	Mean	Av. behav.
$\Phi_{e,B02}$ [‐]	*0.20*	*0.20*	*0.20*	*0.20*	*0.20*	*0.20*	*0.20*	*0.20*	*0.20*	*0.20*	*0.20*	*0.20*	*0.20*	*0.20*	*0.20*	*0.20*	*0.20*	*0.20*	*0.20*	***0.20***	***0.20***
$\Phi_{e,H29}$ [‐]	0.24	0.24	0.21	0.19	0.14	0.18	0.18	0.19	0.25	0.28	0.19	0.27	0.14	0.21	0.15	0.15	0.23	0.22	0.18	**0.20**	**0.20**
$\Phi_{e,H52}$ [‐]	0.23	0.23	0.21	0.22	0.18	0.16	0.17	0.25	0.26	0.24	0.19	0.16	0.16	0.19	0.18	0.21	0.24	0.20	0.22	**0.21**	**0.21**
$\Phi_{e,R12}$ [‐]	0.22	0.27	0.20	0.21	0.17	0.19	0.16	0.24	0.32	0.26	0.32	/	0.18	0.19	0.18	0.20	0.20	0.21	0.22	**0.21**	**0.21**
$\Phi_{e,R30}$ [‐]	0.21	0.20	0.17	0.21	0.16	0.16	0.22	0.23	0.30	0.25	0.16	0.20	0.18	0.20	0.18	0.19	0.24	0.21	0.21	**0.20**	**0.20**
$\alpha_{B02}$ [°]	43.9	41.4	37.4	41.3	37.1	38.4	45.0	36.3	46.1	11.5	47.2	42.9	40.9	47.7	47.1	43.1	34.6	37.3	48.9	**40.4**	**38.2**
$\alpha_{H29}$ [°]	40.1	41.4	46.8	36.6	30.5	37.6	39.8	33.8	44.9	46.2	33.5	47.4	33.8	45.4	30.7	31.5	41.6	40.6	47.7	**39.5**	**39.6**
$\alpha_{H52}$ [°]	45.5	46.0	36.6	32.1	31.4	31.2	30.6	31.5	45.1	40.6	39.5	42.0	35.1	40.6	31.1	41.1	41.3	41.7	51.2	**38.7**	**38.9**
$\alpha_{R12}$ [°]	44.5	44.4	45.8	40.8	30.2	38.6	36.2	47.8	49.7	41.3	35.2	/	47.6	35.0	32.0	36.1	43.4	42.9	49.5	**41.2**	**42.7**
$\alpha_{R30}$ [°]	36.4	44.5	36.4	48.9	38.4	47.0	34.6	35.4	47.0	44.8	32.8	46.0	43.6	36.3	31.7	39.0	42.8	36.6	50.9	**40.7**	**42.3**
$\lambda_{c,B02}$ [‐]	1.11	1.19	1.18	1.11	1.16	1.15	1.12	1.10	1.07	1.08	1.09	1.14	1.16	1.13	1.10	1.14	1.08	1.09	1.10	**1.12**	**1.14**
$\lambda_{c,H29}$ [‐]	1.10	1.20	1.21	1.11	1.12	1.16	1.09	1.09	1.07	1.12	1.07	1.17	1.13	1.14	1.07	1.10	1.08	1.09	1.07	**1.12**	**1.14**
$\lambda_{c,H52}$ [‐]	1.11	1.21	1.20	1.13	1.15	1.14	1.10	1.11	1.08	1.10	1.06	1.12	1.14	1.13	1.08	1.13	1.09	1.09	1.12	**1.12**	**1.14**
$\lambda_{c,R12}$ [‐]	1.10	1.25	1.21	1.10	1.14	1.15	1.09	1.12	1.13	1.10	1.07	/	1.15	1.12	1.08	1.11	1.09	1.09	1.12	**1.12**	**1.15**
$\lambda_{c,R30}$ [‐]	1.11	1.20	1.18	1.14	1.14	1.17	1.10	1.11	1.11	1.10	1.07	1.16	1.15	1.12	1.08	1.13	1.10	1.10	1.10	**1.12**	**1.15**
$\lambda_{\mathrm{VSMC},B02}$ [‐]	1.46	1.20	1.18	1.20	1.26	1.50	1.20	1.24	1.05	1.50	1.08	1.31	1.25	1.28	1.50	1.36	1.50	1.50	1.03	**1.29**	**1.21**
$\lambda_{\mathrm{VSMC},H29}$ [‐]	1.38	1.15	1.52	1.13	1.43	1.31	0.93	1.44	1.31	1.17	1.03	1.46	1.42	1.53	1.33	1.41	1.48	1.19	0.96	**1.30**	**1.15**
$\lambda_{\mathrm{VSMC},H52}$ [‐]	1.34	1.30	1.19	1.22	1.20	1.46	1.40	1.38	1.14	1.27	0.92	1.46	1.34	1.32	1.23	1.29	1.50	1.07	1.20	**1.28**	**1.18**
$\lambda_{\mathrm{VSMC},R12}$ [‐]	‐	1.43	1.46	1.10	1.33	1.26	1.00	1.46	1.16	1.16	1.00	/	1.49	1.24	1.05	1.16	1.54	1.52	1.05	**1.26**	**1.21**
$\lambda_{\mathrm{VSMC},R30}$ [‐]	1.46	1.52	1.22	1.21	1.14	1.36	1.06	1.20	1.16	1.30	1.04	1.56	1.47	1.12	1.08	1.52	1.56	1.40	1.46	**1.31**	**1.22**
$S_{1,B02}$ [‐]	*0.30*	*0.30*	*0.30*	*0.30*	*0.30*	*0.30*	*0.30*	*0.30*	*0.30*	*0.30*	*0.30*	*0.30*	*0.30*	*0.30*	*0.30*	*0.30*	*0.30*	*0.30*	*0.30*	***0.30***	***0.30***
$S_{1,H29}$ [‐]	0.16	0.46	0.16	0.51	0.10	0.56	1.00	0.14	0.11	0.88	0.38	0.21	0.11	0.40	0.03	0.17	0.22	1.00	0.34	**0.36**	**0.30**
$S_{1,H52}$ [‐]	0.27	0.17	0.51	0.73	0.31	0.30	0.18	0.20	0.34	0.62	0.82	0.03	0.14	0.67	0.28	0.39	0.32	0.93	0.07	**0.38**	**0.30**
$S_{1,R12}$ [‐]	0.00	0.30	0.19	0.07	0.16	0.55	0.49	0.11	0.46	0.58	0.59	/	0.09	0.63	0.75	0.39	0.24	0.27	0.35	**0.35**	**0.24**
$S_{1,R30}$ [‐]	0.52	0.10	0.23	0.28	0.27	0.11	0.55	0.49	0.41	0.48	0.17	0.27	0.14	1.00	0.73	0.36	0.47	0.74	0.02	**0.39**	**0.26**
$\Phi_{\mathrm{VSMC},B02}$ [‐]	0.08	0.39	0.42	0.19	0.48	0.18	0.30	0.43	0.41	0.49	0.62	0.32	0.52	0.18	0.17	0.36	0.13	0.12	0.65	**0.34**	**0.39**
$\Phi_{\mathrm{VSMC},H29}$ [‐]	0.10	0.46	0.44	0.18	0.35	0.17	0.26	0.41	0.51	0.68	0.61	0.43	0.37	0.19	0.13	0.28	0.15	0.13	0.60	**0.33**	**0.39**
$\Phi_{\mathrm{VSMC},H52}$ [‐]	0.10	0.44	0.44	0.21	0.44	0.15	0.25	0.54	0.54	0.59	0.58	0.26	0.41	0.17	0.15	0.37	0.15	0.11	0.72	**0.35**	**0.40**
$\Phi_{\mathrm{VSMC},R12}$ [‐]	0.09	0.53	0.42	0.19	0.42	0.17	0.24	0.51	0.65	0.65	0.65	/	0.46	0.17	0.15	0.35	0.13	0.12	0.72	**0.36**	**0.42**
$\Phi_{\mathrm{VSMC},R30}$ [‐]	0.09	0.39	0.36	0.20	0.40	0.15	0.32	0.48	0.62	0.61	0.51	0.32	0.47	0.18	0.15	0.35	0.15	0.12	0.67	**0.34**	**0.40**

Missing parameters denoted by a ‘/’ indicates that no data was available for a participant at a given time point. Missing VSMC parameters denoted by a ‘‐’ indicate that the model predicted no VSMC contribution at that time point (i.e., $S_{1}=0$), so that relevant parameters cannot be estimated. Average behaviour (Av. behav.) model parameters were obtained by fitting ensemble averaged pressure–diameter relationship across the entire cohort of participants with complete acquisition protocols (i.e., excluding D1 and F2). Italic indicates imposed (non‐fitted) model parameters. Boldfacing was use to highlight average parameter values in the cohort of 20 participants.

**Table A4:** Time‐dependent model parameters of the femoral artery.

Partecipant	A1	A2	B1	B2	C1	C2	D1	D2	E1	E2	F1	F2	G1	G2	H1	H2	I2	J1	J2	Mean	Av. behav.
$\Phi_{e,B02}$ [‐]	*0.20*	*0.20*	*0.20*	*0.20*	*0.20*	*0.20*	*0.20*	*0.20*	*0.20*	*0.20*	*0.20*	*0.20*	*0.20*	*0.20*	*0.20*	*0.20*	*0.20*	*0.20*	*0.20*	***0.20***	***0.20***
$\Phi_{e,H29}$ [‐]	0.10	0.23	0.11	0.43	0.25	0.71	0.21	0.18	0.11	0.30	0.07	0.35	0.16	0.27	0.09	0.17	0.15	0.20	0.12	**0.22**	**0.18**
$\Phi_{e,H52}$ [‐]	0.28	0.11	0.28	0.23	0.26	0.43	0.45	0.19	0.21	0.31	0.19	0.33	0.41	0.20	0.23	0.18	0.16	0.33	0.09	**0.26**	**0.23**
$\Phi_{e,R12}$ [‐]	0.34	0.11	0.17	0.16	0.39	0.28	0.35	0.31	0.15	0.27	0.17	/	0.17	0.42	0.11	0.37	0.18	0.28	0.19	**0.25**	**0.22**
$\Phi_{e,R30}$ [‐]	0.11	0.10	0.10	0.12	0.20	0.28	0.16	0.20	0.07	0.18	0.08	0.20	0.26	0.23	0.11	0.15	0.15	0.17	0.11	**0.16**	**0.15**
$\alpha_{B02}$ [°]	46.9	32.5	47.6	38.7	40.9	24.8	53.2	54.4	48.2	17.6	47.1	32.6	44.2	39.3	44.7	45.0	3.6	57.0	45.9	**40.2**	**47.9**
$\alpha_{H29}$ [°]	44.8	49.6	24.7	22.9	32.3	63.7	44.0	40.1	44.0	43.8	38.2	50.0	44.6	48.7	38.6	26.0	46.9	46.0	44.4	**41.8**	**46.1**
$\alpha_{H52}$ [°]	56.6	65.6	45.7	46.1	45.8	57.0	53.6	19.4	26.1	49.8	20.4	52.8	56.2	49.8	45.5	25.8	71.4	61.2	21.9	**45.8**	**50.9**
$\alpha_{R12}$ [°]	53.7	89.8	27.8	45.2	53.9	52.7	31.6	44.2	41.9	40.3	40.2	/	57.7	57.2	43.3	52.8	79.6	58.7	17.3	**49.3**	**50.5**
$\alpha_{R30}$ [°]	50.4	65.2	51.3	67.8	43.0	47.3	26.4	21.6	90.0	56.4	20.0	42.1	46.4	48.2	39.6	29.0	82.0	35.8	45.9	**47.8**	**42.4**
$\lambda_{c,B02}$ [‐]	1.06	1.08	1.11	1.04	1.09	1.04	1.06	1.05	1.12	1.06	1.08	1.08	1.05	1.11	1.14	1.07	1.09	1.06	1.11	**1.08**	**1.08**
$\lambda_{c,H29}$ [‐]	0.98	1.12	1.08	1.10	1.10	1.53	1.02	1.07	0.98	1.06	0.96	1.20	0.97	1.21	1.06	1.06	0.79	1.04	0.94	**1.07**	**1.05**
$\lambda_{c,H52}$ [‐]	1.19	0.88	1.35	1.01	1.20	1.18	1.30	1.04	1.15	1.14	1.09	1.21	1.30	1.17	1.25	1.07	0.86	1.37	1.05	**1.15**	**1.13**
$\lambda_{c,R12}$ [‐]	1.22	0.76	1.12	0.80	1.25	1.05	1.10	1.04	1.09	1.04	1.08	/	1.12	1.48	1.11	1.24	0.84	1.23	1.09	**1.09**	**1.10**
$\lambda_{c,R30}$ [‐]	0.82	0.79	0.89	0.77	1.09	1.02	1.02	1.03	0.73	0.92	1.03	1.07	1.08	1.13	1.04	1.04	0.77	0.99	0.90	**0.95**	**0.98**
$\lambda_{\mathrm{VSMC},B02}$ [‐]	1.15	1.18	1.09	1.10	1.18	1.45	1.01	1.00	1.12	1.32	1.38	1.49	1.19	1.17	1.22	1.29	1.05	1.00	1.12	**1.19**	**1.23**
$\lambda_{\mathrm{VSMC},H29}$ [‐]	‐	1.03	0.92	1.23	1.24	2.03	0.97	1.21	‐	1.05	‐	1.56	0.85	1.53	0.91	1.04	0.74	0.99	0.84	**1.13**	**1.22**
$\lambda_{\mathrm{VSMC},H52}$ [‐]	1.77	0.83	1.51	1.43	1.53	1.58	1.49	1.26	1.21	1.33	1.50	1.67	1.46	1.32	1.34	1.13	0.89	1.38	0.85	**1.34**	**1.38**
$\lambda_{\mathrm{VSMC},R12}$ [‐]	1.43	0.76	1.19	0.71	1.54	1.17	1.42	0.98	1.03	0.98	1.25	/	1.20	1.44	0.98	1.28	‐	1.34	0.98	**1.16**	**1.20**
$\lambda_{\mathrm{VSMC},R30}$ [‐]	‐	0.78	‐	0.75	1.03	1.04	‐	1.35	0.71	‐	1.10	0.94	1.17	1.06	0.86	‐	0.76	0.88	0.83	**0.95**	**0.96**
$S_{1,B02}$ [‐]	*0.30*	*0.30*	*0.30*	*0.30*	*0.30*	*0.30*	*0.30*	*0.30*	*0.30*	*0.30*	*0.30*	*0.30*	*0.30*	*0.30*	*0.30*	*0.30*	*0.30*	*0.30*	*0.30*	***0.30***	***0.30***
$S_{1,H29}$ [‐]	0.00	0.95	1.00	0.75	0.50	0.92	0.55	0.77	0.00	0.73	0.00	0.71	0.25	0.42	1.00	0.91	0.78	0.77	0.33	**0.60**	**0.42**
$S_{1,H52}$ [‐]	0.16	1.00	1.00	0.14	0.78	1.00	0.98	0.27	0.89	0.43	0.89	0.53	0.75	0.43	1.00	1.00	0.25	1.00	0.13	**0.66**	**0.82**
$S_{1,R12}$ [‐]	0.41	1.00	1.00	1.00	0.24	0.70	0.86	1.00	1.00	0.76	0.75	/	0.13	0.74	0.74	1.00	0.00	0.63	1.00	**0.72**	**0.93**
$S_{1,R30}$ [‐]	0.00	1.00	0.00	1.00	1.00	0.70	0.00	0.05	1.00	0.00	0.12	1.00	0.74	0.51	1.00	0.00	1.00	0.52	0.37	**0.53**	**0.10**
$\Phi_{\mathrm{VSMC},B02}$ [‐]	0.39	0.66	0.51	0.25	0.26	0.08	0.25	0.42	0.70	0.37	0.66	0.30	0.29	0.28	0.66	0.33	0.80	0.40	0.79	**0.44**	**0.16**
$\Phi_{\mathrm{VSMC},H29}$ [‐]	0.20	0.76	0.29	0.54	0.32	0.29	0.26	0.38	0.39	0.56	0.23	0.53	0.23	0.38	0.28	0.28	0.61	0.40	0.47	**0.39**	**0.15**
$\Phi_{\mathrm{VSMC},H52}$ [‐]	0.55	0.36	0.72	0.29	0.34	0.17	0.55	0.41	0.73	0.58	0.64	0.50	0.59	0.28	0.76	0.29	0.64	0.67	0.37	**0.50**	**0.19**
$\Phi_{\mathrm{VSMC},R12}$ [‐]	0.66	0.36	0.43	0.20	0.52	0.11	0.43	0.66	0.53	0.50	0.57	/	0.24	0.58	0.37	0.61	0.72	0.55	0.73	**0.49**	**0.19**
$\Phi_{\mathrm{VSMC},R30}$ [‐]	0.22	0.34	0.26	0.16	0.26	0.11	0.20	0.42	0.25	0.33	0.27	0.31	0.37	0.32	0.36	0.24	0.59	0.34	0.43	**0.31**	**0.12**

Missing parameters denoted by a ‘/’ indicates that no data was available for a participant at a given time point. Missing VSMC parameters denoted by a ‘‐’ indicate that the model predicted no VSMC contribution at that time point (i.e., $S_{1}=0$), so that relevant parameters cannot be estimated. Average behaviour (Av. behav.) model parameters were obtained by fitting ensemble averaged pressure–diameter relationship across the entire cohort of participants with complete acquisition protocols (i.e., excluding D1 and F2). Italic indicates imposed (non‐fitted) model parameters. Boldfacing was use to highlight average parameter values in the cohort of 20 participants.

**Table A5:** Time‐dependent model parameters of the popliteal artery.

Partecipant	A1	A2	B1	B2	C1	C2	D1	D2	E1	E2	F1	F2	G1	G2	H1	H2	I2	J1	J2	Mean	Av. behav.
$\Phi_{e,B02}$ [‐]	*0.20*	*0.20*	*0.20*	*0.20*	*0.20*	*0.20*	*0.20*	*0.20*	*0.20*	*0.20*	*0.20*	*0.20*	*0.20*	*0.20*	*0.20*	*0.20*	*0.20*	*0.20*	*0.20*	***0.20***	***0.20***
$\Phi_{e,H29}$ [‐]	0.15	0.18	0.18	0.19	0.33	0.26	0.15	0.18	0.21	0.17	0.20	0.22	0.16	0.17	0.23	0.15	0.26	0.16	0.21	**0.20**	**0.19**
$\Phi_{e,H52}$ [‐]	0.16	0.22	0.18	0.17	0.27	0.16	0.14	0.20	0.23	0.18	0.18	0.33	0.21	0.19	0.25	0.18	0.23	0.22	0.36	**0.21**	**0.21**
$\Phi_{e,R12}$ [‐]	0.15	0.14	0.18	0.13	0.16	0.15	/	0.15	0.25	0.20	0.18	/	0.14	0.17	0.23	0.18	0.23	0.20	0.24	**0.18**	**0.18**
$\Phi_{e,R30}$ [‐]	0.13	0.14	0.19	0.14	0.24	0.25	0.16	0.24	0.20	0.21	0.16	0.20	0.13	0.24	0.21	0.17	0.26	0.21	0.25	**0.19**	**0.19**
$\alpha_{B02}$ [°]	48.2	52.7	45.5	42.9	40.4	47.4	45.9	50.9	51.9	23.2	89.8	45.6	44.6	44.6	53.7	43.3	44.1	44.1	90.0	**49.9**	**52.5**
$\alpha_{H29}$ [°]	59.2	44.0	73.3	44.5	53.0	46.0	52.1	48.0	53.7	48.6	24.8	56.3	47.2	45.9	54.6	37.6	18.5	34.8	39.6	**46.4**	**49.2**
$\alpha_{H52}$ [°]	21.5	55.3	52.2	17.9	49.5	25.3	26.8	20.5	46.4	52.2	57.0	57.2	37.0	42.9	54.9	37.5	45.8	29.6	59.3	**41.5**	**47.2**
$\alpha_{R12}$ [°]	49.5	28.5	43.8	39.5	36.6	38.4	/	32.4	41.0	35.7	50.6	/	45.0	40.6	56.1	45.9	46.2	42.1	46.6	**42.3**	**47.3**
$\alpha_{R30}$ [°]	44.4	46.5	36.1	48.2	55.4	38.9	47.6	49.2	51.2	49.4	38.4	52.7	25.1	56.7	51.6	49.6	45.3	37.9	59.6	**46.5**	**51.9**
$\lambda_{c,B02}$ [‐]	1.09	1.12	1.09	1.10	1.08	1.08	1.10	1.08	1.08	1.17	1.09	1.08	1.12	1.13	1.07	1.13	1.05	1.13	1.06	**1.10**	**1.10**
$\lambda_{c,H29}$ [‐]	0.98	1.13	1.30	1.06	1.30	1.17	1.07	1.09	1.15	1.19	1.17	1.21	1.14	1.18	1.13	1.12	1.10	1.12	1.11	**1.14**	**1.14**
$\lambda_{c,H52}$ [‐]	1.06	1.31	1.14	1.10	1.25	1.07	1.06	1.08	1.13	1.21	1.12	1.38	1.14	1.23	1.20	1.15	1.12	1.20	1.45	**1.18**	**1.16**
$\lambda_{c,R12}$ [‐]	0.99	1.10	1.08	1.01	1.08	1.00	/	1.04	1.13	1.18	1.13	/	1.11	1.14	1.15	1.14	1.09	1.17	1.08	**1.09**	**1.10**
$\lambda_{c,R30}$ [‐]	0.92	1.13	1.07	1.11	1.19	1.09	1.01	1.08	1.07	1.21	1.06	1.13	1.08	1.42	1.04	1.14	1.11	1.16	1.19	**1.12**	**1.11**
$\lambda_{\mathrm{VSMC},B02}$ [‐]	1.04	1.01	1.04	1.11	1.08	1.07	1.07	1.01	1.00	1.44	1.01	1.03	1.06	1.02	1.01	1.06	1.05	1.00	1.00	**1.06**	**1.00**
$\lambda_{\mathrm{VSMC},H29}$ [‐]	0.91	1.11	1.21	0.94	1.40	1.30	1.56	1.09	1.15	1.66	1.41	1.37	1.11	1.22	1.12	1.22	1.31	1.16	1.26	**1.24**	**1.19**
$\lambda_{\mathrm{VSMC},H52}$ [‐]	1.01	1.54	1.09	0.99	1.44	1.06	1.03	1.27	1.13	1.15	1.09	1.88	1.12	1.39	1.21	1.24	1.20	1.35	1.74	**1.26**	**1.26**
$\lambda_{\mathrm{VSMC},R12}$ [‐]	0.91	1.04	1.01	0.84	1.51	0.85	/	0.94	1.06	1.14	1.13	/	1.06	1.09	1.17	1.44	1.11	1.17	1.11	**1.09**	**1.05**
$\lambda_{\mathrm{VSMC},R30}$ [‐]	‐	1.10	1.00	1.08	1.59	1.04	0.91	1.04	‐	1.08	1.39	1.62	0.97	1.57	‐	1.06	1.09	1.12	1.26	**1.18**	**1.14**
$S_{1,B02}$ [‐]	*0.30*	*0.30*	*0.30*	*0.30*	*0.30*	*0.30*	*0.30*	*0.30*	*0.30*	*0.30*	*0.30*	*0.30*	*0.30*	*0.30*	*0.30*	*0.30*	*0.30*	*0.30*	*0.30*	***0.30***	***0.30***
$S_{1,H29}$ [‐]	1.00	1.00	1.00	0.41	1.00	0.50	0.01	0.73	0.62	0.03	0.57	0.99	1.00	0.59	0.62	0.66	0.42	0.71	0.93	**0.67**	**0.94**
$S_{1,H52}$ [‐]	0.84	0.20	0.96	0.99	1.00	0.91	0.84	0.44	0.84	0.94	0.28	0.79	0.98	0.76	0.71	0.88	0.85	0.81	0.95	**0.79**	**0.97**
$S_{1,R12}$ [‐]	0.91	0.99	0.72	0.78	0.07	1.00	/	1.00	0.99	1.00	0.99	/	0.16	0.95	0.38	0.11	0.73	0.92	1.00	**0.75**	**1.00**
$S_{1,R30}$ [‐]	0.00	0.47	0.51	0.66	0.12	1.00	1.00	0.23	0.00	0.98	0.08	0.11	1.00	0.32	0.00	0.68	0.98	1.00	0.99	**0.53**	**0.25**
$\Phi_{\mathrm{VSMC},B02}$ [‐]	0.79	0.70	0.78	0.65	0.36	0.53	0.78	0.56	0.59	0.54	0.78	0.35	0.64	0.62	0.61	0.65	0.57	0.72	0.35	**0.61**	**0.32**
$\Phi_{\mathrm{VSMC},H29}$ [‐]	0.61	0.61	0.69	0.60	0.59	0.70	0.59	0.49	0.63	0.45	0.80	0.38	0.51	0.52	0.70	0.49	0.74	0.58	0.38	**0.58**	**0.31**
$\Phi_{\mathrm{VSMC},H52}$ [‐]	0.61	0.78	0.69	0.54	0.48	0.42	0.55	0.58	0.69	0.49	0.69	0.58	0.66	0.58	0.75	0.58	0.67	0.78	0.64	**0.62**	**0.33**
$\Phi_{\mathrm{VSMC},R12}$ [‐]	0.59	0.48	0.69	0.41	0.29	0.41	/	0.44	0.74	0.54	0.71	/	0.45	0.52	0.72	0.58	0.65	0.73	0.42	**0.55**	**0.29**
$\Phi_{\mathrm{VSMC},R30}$ [‐]	0.51	0.49	0.72	0.45	0.43	0.67	0.61	0.68	0.58	0.56	0.63	0.36	0.41	0.74	0.64	0.54	0.73	0.75	0.44	**0.57**	**0.30**

Missing parameters denoted by a ‘/’ indicates that no data was available for a participant at a given time point. Missing VSMC parameters denoted by a ‘‐’ indicate that the model predicted no VSMC contribution at that time point (i.e., $S_{1}=0$), so that relevant parameters cannot be estimated. Average behaviour (Av. behav.) model parameters were obtained by fitting ensemble averaged pressure–diameter relationship across the entire cohort of participants with complete acquisition protocols (i.e., excluding D1 and F2). Italic indicates imposed (non‐fitted) model parameters. Boldfacing was use to highlight average parameter values in the cohort of 20 participants.
