# Supplementary material for: A constitutive modelling framework for applications to in vivo longitudinal data: Evaluation in a 60‐day head‐down bed rest study on arterial function
Source: J Physiol. 2026 Mar 24;604(19):8028–49. doi: 10.1113/JP289863 (PMC13628561; doi:10.1113/JP289863)
Supplement: Supplementary file 1 [file TJP-604-8028-s001.pdf]

The Journal of Physiology

A constitutive modelling framework for applications to *in vivo* longitudinal data: Evaluation in 60-day head-down bed rest study on arterial function

Alessandro Giudici, Karen Barchetti, Umit Gencer, Hakim Khettab, Elie Mousseaux, Carole Leguy, Tammo Delhaas, Rosa Maria Bruno, Bart Spronck, and Pierre Boutouyrie
DOI: 10.1113/JP289863

Corresponding author(s): Alessandro Giudici (a.giudici@maastrichtuniversity.nl)

The following individual(s) involved in review of this submission have agreed to reveal their identity: Stephane Avril (Referee #2); Pasquale Ciarletta (Referee #3)

Review Timeline:

Submission Date:	02-Sep-2025
Editorial Decision:	21-Oct-2025
Revision Received:	21-Nov-2025
Accepted:	21-Jan-2026

Senior Editor: Eleonora Grandi

Reviewing Editor: Christian Vergara

Transaction Report:

(Note: With the exception of the correction of typographical or spelling errors that could be a source of ambiguity, letters and reports are not edited. Depending on transfer agreements, referee reports obtained elsewhere may or may not be included in this compilation. Referee reports are anonymous unless the Referee chooses to sign their reports.)

Re: JP-RP-2025-289863 "**A constitutive modelling framework for applications to *in vivo* longitudinal data: Evaluation in 60 day head-down bed rest study on arterial function**" by Alessandro Giudici, Karen Barchetti, Umit Gencer, Hakim Khettab, Elie Mousseaux, Carole Leguy, Tammo Delhaas, Rosa Maria Bruno, Bart Spronck, and Pierre BOUTOUYRIE

Dear Dr Giudici,

Thank you for submitting your manuscript to The Journal of Physiology. It has been assessed by a Reviewing Editor and by 2 expert referees and we are pleased to tell you that it is potentially acceptable for publication following satisfactory major revision.

Please advise your co-authors of this decision as soon as possible.

The referee reports are copied at the end of this email.

Please address all the points raised and incorporate all requested revisions or explain in your Response to Referees why a change has not been made. We hope you will find the comments helpful and that you will be able to return your revised manuscript within 2 months. If your article is NOT for a Special Issue, you may have 9 months to revise. If you require an extension, please contact journal staff: jp@physoc.org. Please note that this letter does not constitute a guarantee for acceptance of your revised manuscript.

Your revised manuscript should be submitted online using the link in your Author Tasks: Link Not Available. This link is accessible via your account as Corresponding Author; it is not available to your co-authors. If this presents a problem, please contact journal staff (jp@physoc.org). Image files from the previous version are retained on the system. Please ensure you replace or remove any files that are being revised.

If you do not wish to submit a revised version of your manuscript, you must inform our journal staff (jp@physoc.org) or reply to this email to request withdrawal. Please note that a manuscript must be formally withdrawn from the peer review process at one journal before it may be submitted to another journal.

TRANSPARENT PEER REVIEW POLICY: To improve the transparency of its peer review process, The Journal of Physiology publishes online as supporting information the peer review history of all articles accepted for publication. Readers will have access to decision letters, including Editors' comments and referee reports, for each version of the manuscript, as well as any author responses to peer review comments. Referees can decide whether or not they wish to be named on the peer review history document.

ABSTRACT FIGURES: Authors are expected to use The Journal's premium BioRender account to create/redraw their Abstract Figures. Information on how to access this account is here: https://physoc.onlinelibrary.wiley.com/journal/14697793/biorender-access.

This will enable Authors to create and download high-resolution figures. If authors have used the free BioRender service, they can use the instructions provided in the link above to download a high-resolution version suitable for publication.

The link provided should only be used for the purposes of this submission. Authors will be charged for figures created on this account if they are not related to this manuscript submission.

LANGUAGE EDITING AND SUPPORT FOR PUBLICATION: If you would like help with English language editing, or other article preparation support, Wiley Editing Services offers expert help, including English Language Editing, as well as translation, manuscript formatting, and figure formatting at www.wileyauthors.com/eeo/preparation. You can also find resources for Preparing Your Article for general guidance about writing and preparing your manuscript at www.wileyauthors.com/eeo/prepresources.

RIGOUR AND REPRODUCIBILITY: We recommend authors consult The Journal's Rigour and Reproducibility requirements to ensure that their manuscript is suitable for publication: https://physoc.onlinelibrary.wiley.com/pb-assets/hub-assets/physoc/documents/TJP-Rigour-and-Reproducibility-Requirements.pdf

REVISION CHECKLIST:

Check that your Methods section conforms to journal policy: https://jp.msubmit.net/cgi-bin/main.plex?form_type=display_requirements#methods.

Check that data presented conforms to the statistics policy: https://jp.msubmit.net/cgi-bin/main.plex?form_type=display_requirements#statistics.

Upload a full Response to Referees file. To create your 'Response to Referees': copy all the reports, including any comments from the Senior and Reviewing Editors, into a Microsoft Word, or similar, file and respond to each point, using font or background colour to distinguish comments and responses and upload as the required file type.

Please upload two versions of your manuscript text: one with all relevant changes highlighted and one clean version with no changes tracked. The manuscript file should include all tables and figure legends, but each figure/graph should be uploaded as separate, high-resolution files.

You may also upload:

- 'Potential Cover Art' for consideration as the issue's cover image
- Appropriate Supporting Information (Video, audio or data set: see https://jp.msubmit.net/cgi-bin/main.plex?form_type=display_requirements#supp).

We look forward to receiving your revised submission.

If you have any queries, please reply to this email and we will be pleased to advise.

Yours sincerely,

Eleonora Grandi
Senior Editor
The Journal of Physiology

REQUIRED ITEMS

- Please upload separate high-quality figure files via the submission form.

EDITOR COMMENTS

Reviewing Editor:

Please carefully address the remarks highlighted by the Referees, detailing your answers in a dedicated letter, reporting lines/pages of modifications and using colours for the changed/added parts in the new manuscript.

Senior Editor:

Comments to the Author:
Please address the reviewers' suggestions, with careful attention to including greater detail and justification of model assumptions (and quantification through sensitivity).

REFEREE COMMENTS

Referee #1:

The authors proposed an original methodological development that enhances the understanding and quantification of underlying microstructural mechanisms of arterial remodeling following head-down bedrest (HDBR) situation, with results showing increased VSMC contraction and microstructural re-arrangement of collagen fibres as key adaptations to haemodynamic changes during HDBR, also resulting in reversible de-stiffening of peripheral arteries.

The paper is well written, with clear presentation of methodology and results. The authors provide valuable insights into the underlying mechanisms of vascular adaption during HDBR, making a meaningful contribution to the understanding of the problem.

In summary, the manuscript presents an interesting study with potential impact. However, some clarification on certain points is needed and I recommend a major revision before publication, involving addressing the following comments :

1. compared to Masson et al 2011, where a similar methodology was proposed, here the authors have extensively discussed all measured taken to reduce the number of unknown parameters and avoid non unique solutions to the identification problem. However, it would be interesting in the result to show that these measures have paid, for instance by showing that starting from n=100 initial guesses of model parameters always lead to the same unique solution.

2. Eq. 12 is valid for a long straight tube, how much is this valid for the poplital artery ? Moreover, using Eq. 19 in Eq. 13 is just another way of using the pressure diameter recordings, as the PVWext is not measured but estimated from systolic and diastolic pressures and diameters. Have you tried Eq. 13 without PVW ? Please report the weights wP, wF and wPVW of Eq. 13.

3. Table 1, please provide references for the choice of fit ranges and for parameters coming from literature.

4. the authors acknowledge in the discussion that they calculated the avearge circumferential stresses at a uniform pressure of 100 mmHg without accounting for expected/potential differences in average pressure exposures between different time points and arterial sites. However the actual blood pressures are known (see data in Fig A1) so why using 100mmHg instead of the actual pressure ?

5. the authors acknowledge that the main limitation of their approach is to neglect the adventitia in the mechanical response of arteries. However, quantitative analysis of stress distribution between media and adventitia should be provided to support this assumption, assessing how much is the fraction of stress taken by the adventitia in these arteries, otherwise all the discussion on how much stress distributes in VSMC or elastin is not acceptable

6. the conclusion of all the dicussion is that remodelling observed in the femoral and poplital arteries may be more driven by WSS than wall stress. The authors say they expect a reduction of WSS in the femoral and poplital arteries during HDBR and increase in recovery. Are there data supporting that ?

Referee #2:

Please see Referee 2's comments in the attached file.

END OF COMMENTS

The manuscript presents a technically well-developed and ambitious contribution addressing a longstanding challenge in arterial biomechanics: the in vivo parameterization of complex constitutive models. The authors propose a modelling framework that exploits longitudinal and multi-site data to reduce parameter identifiability issues that have so far prevented the clinical use of constrained mixture models. The formulation is rigorous, drawing from the classical foundations of finite elasticity and mixture theory, and the authors provide a detailed and transparent derivation of the constitutive equations, boundary conditions, and parameter estimation strategy.

The main strength of this work lies in its methodological innovation. The coupling of longitudinal measurements with cross-site consistency constraints is a sound and original way to improve parameter identifiability without oversimplifying the underlying physics. The application to the 60-day head-down bed rest dataset is compelling, as it allows the authors to evaluate physiological remodeling under controlled haemodynamic unloading. The quantitative agreement between model predictions and experimental data is satisfactory, with R^2 values around 0.9, and the analysis convincingly isolates the roles of smooth muscle contraction and collagen microstructural rearrangement in arterial adaptation.

The mathematical framework is clearly described and appropriately justified. The assumptions on incompressibility, thin-walled geometry, and fixed axial stretch are consistent with the physiological context. The constrained mixture model with distinct elastin, collagen, and smooth muscle contributions follows the tradition of Humphrey's and Holzapfel's formulations but introduces an elegant dimensionality reduction by imposing inter-arterial and temporal regularity. The optimization strategy is complex but carefully controlled, and the use of reduced axial force and pulse wave velocity as additional constraints is well motivated.

However, some aspects would benefit from deeper clarification. The choice of the reference configuration, defined at mean pressure at baseline, may influence the interpretation of deposition stretches and load-bearing ratios across time; a sensitivity analysis could test the robustness of the inferred microstructural trends. The assumption of constant constituent stiffnesses and volume fractions (except collagen) is physiologically plausible for the study duration but remains a major source of potential bias if applied to other datasets. Moreover, while the longitudinal data improve identifiability, the risk of parameter compensation among Φ and stiffness-like parameters persists, and the authors might discuss the identifiability limits more quantitatively (e.g., by reporting confidence intervals or exploring the cost-function landscape).

From a physiological standpoint, the model offers meaningful insights into vascular remodeling under microgravity analogs. The prediction of reversible peripheral de-stiffening through increased smooth muscle tone and collagen realignment is consistent with experimental evidence and mechanobiological reasoning. Yet, the discussion could more explicitly contrast the simulated wall stress homeostasis with actual pressure exposure changes during bed rest, which may not be uniform across arteries.

Overall, the paper is technically sound, methodologically original, and physiologically relevant. It bridges a critical gap between ex vivo constitutive modelling and clinical applications. Provided that the authors clarify the treatment of the reference configuration, discuss identifiability robustness, and acknowledge the limits of parameter invariance assumptions, the work merits publication. It represents a substantial step toward quantitative phenotyping of arterial remodeling from non-invasive in vivo data.

A constitutive modelling framework for applications to *in vivo* longitudinal data: Evaluation in 60-day head-down bed rest study on arterial function

Alessandro Giudici[a,b], Karen Barchetti[c], Umit Gencer[c,d], Hakim Khettab[c,d], Elie Mousseaux[c,d], Carole Leguy[e,f], Tammo Delhaas[a], Rosa Maria Bruno[c,d], Bart Spronck[a,g,]*, Pierre Boutouyrie[c,d,]*

Response to the reviewer comments

We thank the Reviewers for the time spent reviewing and providing constructive feedback on our manuscript. Below is a point-by-point answer to their individual concerns, with Reviewer comments denoted by the black font, our answers denoted by the blue font, and textual changes in the manuscript denoted by the red font (both in this document and in the revised manuscript).

Reviewer 1

The authors proposed an original methodological development that enhances the understanding and quantification of underlying microstructural mechanisms of arterial remodeling following head-down bedrest (HDBR) situation, with results showing increased VSMC contraction and microstructural re-arrangement of collagen fibres as key adaptations to haemodynamic changes during HDBR, also resulting in reversible de-stiffening of peripheral arteries.

The paper is well written, with clear presentation of methodology and results. The authors provide valuable insights into the underlying mechanisms of vascular adaption during HDBR, making a meaningful contribution to the understanding of the problem.

We thank the reviewer for their overall positive assessment of our manuscript. Below, we provide a point-by-point reply to their questions and remarks.

In summary, the manuscript presents an interesting study with potential impact. However, some clarification on certain points is needed and I recommend a major revision before publication, involving addressing the following comments :

1. compared to Masson et al 2011, where a similar methodology was proposed, here the authors have extensively discussed all measured taken to reduce the number of unknown parameters and avoid non unique solutions to the identification problem. However, it would be interesting in the result to show that these measures have paid, for instance by showing that starting from n=100 initial guesses of model parameters always lead to the same unique solution.

We thank the reviewer for their suggestion. Our choice of repeating the parameter estimation from 100 different initial parameter guesses did not aim to demonstrate that most of these iterations converged to the same solution. Indeed, with 72 fitted model parameters, we cannot expect the cost function to be convex in this 72-dimension space, and most of these iterations will converge to a local

minimum. On the contrary, the *MultiStart* function of MATLAB was used to increase the likelihood of identifying a solution that corresponds to the global minimum of the cost function. We evaluated whether 100 initial parameter guesses are, indeed, enough to reliably converge to the global minimum. To do so, we repeated the parameter estimation for varying number of initial parameter guesses, ranging 1 to 1000. As shown in Figure R1, starting from 20 initial parameter guesses, our parameter estimation consistently converged to the same solution. As such, we can be confident that the 100 initial parameter guesses used in this work allowed for identifying a solution that corresponds to the global minimum.

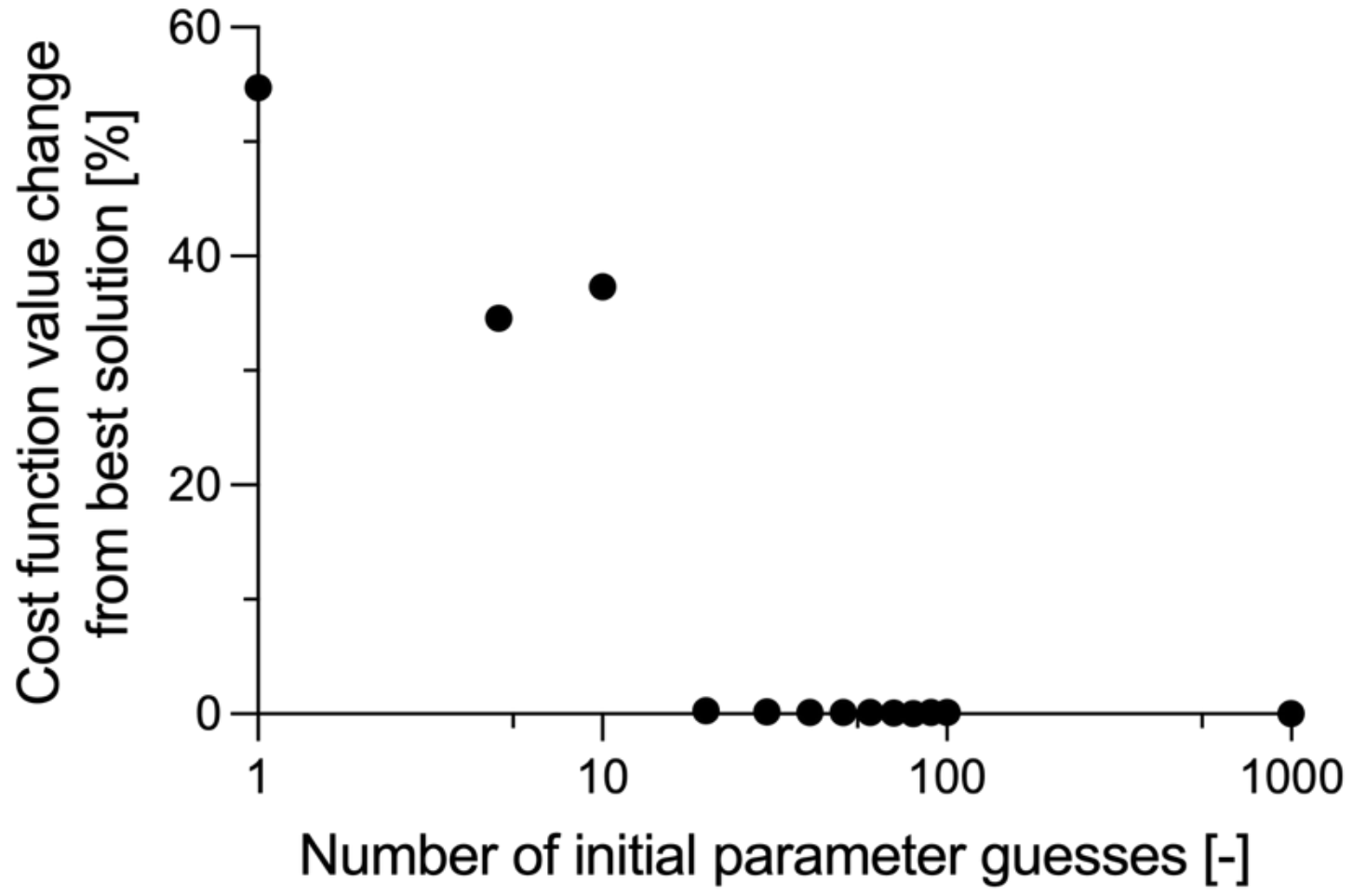

Figure R1 – Identification of the global minimum through the *MultiStart* function of MATLAB. The graph shows that starting from *n*=20 initial parameter guesses, the parameter estimation converged to same solution, indicating with a good level of confidence that the global minimum was successfully identified. The change in cost function value is expressed as percent different with respect to the best solution when evaluating *n*=1000 initial guesses.

We clarified this point in the methods (Page 14, bottom half):

> Our preliminary analysis indicated that *n*=100 initial guesses of model parameters allowed for a consistent and reliable identification of the cost function global minimum.

2. Eq. 12 is valid for a long straight tube, how much is this valid for the poplital artery? Moreover, using Eq. 19 in Eq. 13 is just another way of using the pressure diameter recordings, as the PVWext is not measured but estimated from systolic and diastolic pressures and diameters. Have you tried Eq. 13 without PVW? Please report the weights wP, wF and wPVW of Eq. 13.

We thank the reviewer for their remark and question. Indeed, the Bramwell-Hill equation (Eq. 12) is, in principle, valid only for long straight tubes, and whether, overall, the arteries in our body can be considered long straight tubes is certainly debatable. Nonetheless, in the parameter estimation, pulse wave velocity (PWV) is simply used as an indicator of the slope of the pressure–diameter relationship. It may seem intuitive to assume that including PWV in the cost function (Eq. 13) may be redundant, since PWV is derived from the pressure–diameter data which is already part of the cost function (the first term on the right side of Eq.13). However, the effects of the pressure–diameter and PWV terms are slightly different. The PWV term only penalises based on the slope of the pressure–diameter relationship. Conversely, the pressure – diameter term will quantify the overall matching between the modelled and measured pressure–diameter relationship. As such, a set of model parameters that results in pressure–diameter relationship that runs parallel to the measured

relationship will result in a null PWV error term, despite a non-null error in the pressure–diameter fitting. In our opinion, between two pressure–diameter relationships with a similar mismatch with the measured data, the one with the most similar slope should be considered as a "better fit" (**Figure R2**). We opted for this formulation of the cost function (which we also implemented in other experimental work, e.g., (1)) because in previous fitting attempts where we did not include the PWV term of Eq. 13, best fit solutions would often results in pressure–diameter relationships that did not capture well slopes. Indeed, we believe that the slope carries relevant biomechanical information for the constituent-specific parametrisation.

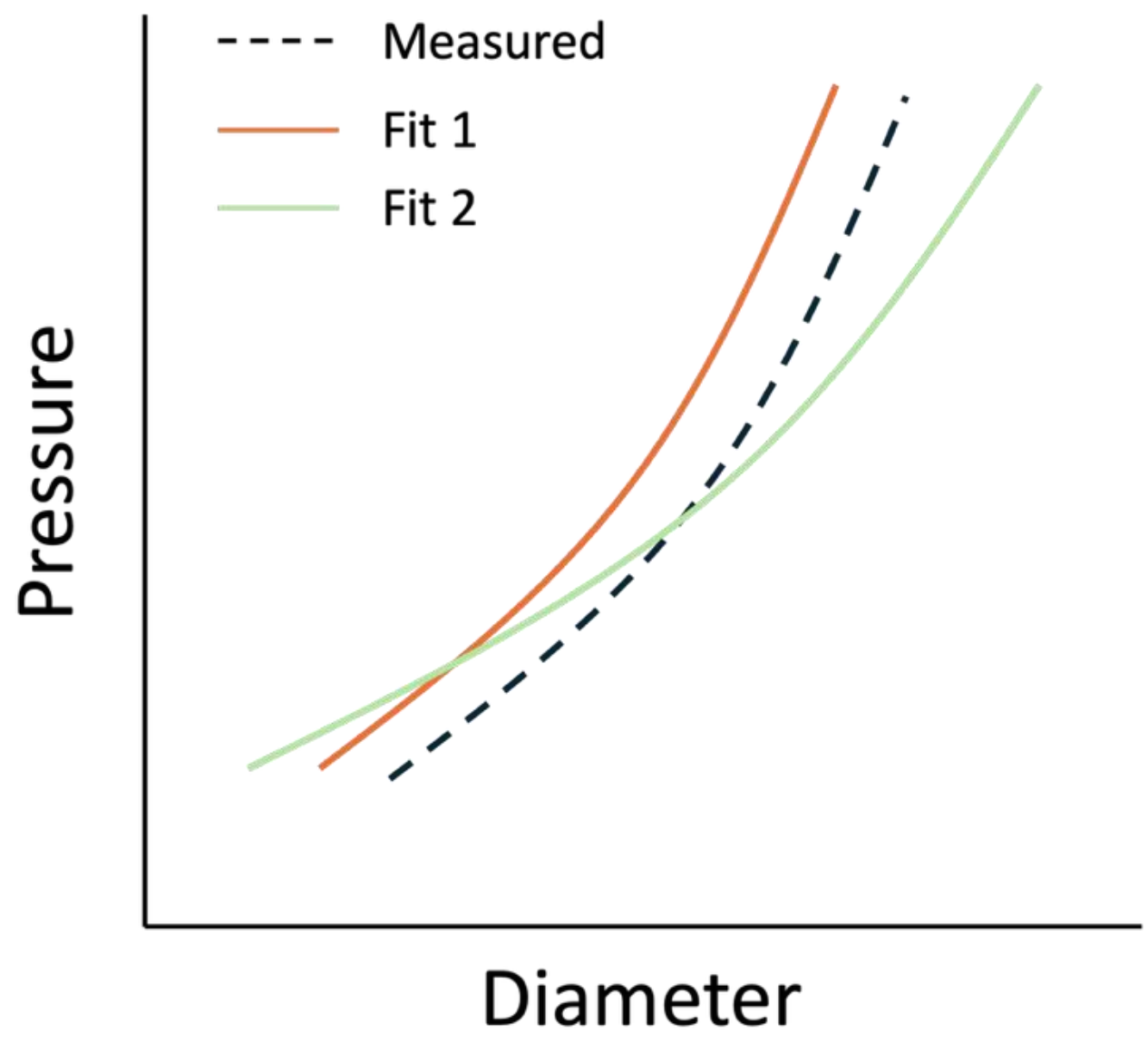

Figure R2 – Comparison of two fitting lines. Fit 1 and Fit 2 are, on average, equally "distant" from the measured data (in terms of squared error), resulting in a similar pressure–diameter error term in the cost function and indicating that the two sets of model parameters are capturing the data equally well. However, the slope of the Fit 1 curve is much closer to that of the measured data. Therefore, Fit 1 should be preferred over Fit 2, which is guaranteed by the PWV term in the cost function.

We clarified this point in the manuscript text:

Page 9, bottom to Page 13, top half:

> *Finally, with our model, we aimed to capture well changes in stiffness between different time points (i.e., the slope of the pressure–diameter relationship). As such, the cost function included also local PWV, defined as*
>
> $$\mathrm{PWV} = d_{\mathrm{d}}\sqrt{\frac{\mathrm{SBP}-\mathrm{DBP}}{\rho\left(d_{\mathrm{s}}^2-d_{\mathrm{d}}^2\right)}}\,,\#(12)$$
>
> *where SBP and DBP are the systolic and diastolic blood pressure, d_s is the lumen diameter at peak systole, and ρ=1050 kg/m^3 is the blood mass density. Notably, inclusion of PWV in the cost function ensures that in case two parameter solutions yield similar matching of the measured pressure–diameter relationship, the one with the closest slope is considered the best fit of the data.*

Finally, the weights of Eq. 13 are provided at the top of page 14:

Lastly, we set $w^P = 1$, $w^F = 1/10$ and $w^{PWV} = N/5$ to achieve a 10:1:2 weighting between the pressure, force and PWV terms in Eq. 13.

3. Table 1, please provide references for the choice of fit ranges and for parameters coming from literature.

We thank the reviewer for their comment. We have added references for the literature-based model parameters. As explained in the manuscript text (page 9, second paragraph), the axial deposition stretch of elastin was assumed to equal the *in vivo* axial stretch of the whole artery, as provided in the work of Learoyd and Taylor (2). In their work, Learoyd and Taylor measured the retraction in axial length of different arteries upon excision, which corresponded to axial stretch of 1.22 and 1.32 for the carotid and femoral artery, respectively. This assumption, therefore, ensures that elastin is axially unloaded upon full retraction after excision from the body. Furthermore, we assumed elastin's deformation field to be equibiaxial when loaded at mean physiological pressure (i.e., 100 mmHg) and *in vivo* axial stretch, thereby equalling the circumferential and the axial deposition stretches. The 20% volume fraction of elastin was assumed based on data of Cox, who reported a 20–35% share of artery dry weight for elastin in several large arteries.

Concerning the ranges of the other model parameters, these were not chosen based on literature but rather deliberately set to allow for large parameter freedom (excluding parameters for which a clear upper and/or lower boundary exists, e.g., the fibre angle α, or the lower bound of all stiffness like model parameters).

4. the authors acknowledge in the discussion that they calculated the avearge circumferential stresses at a uniform pressure of 100 mmHg without accounting for expected/potential differences in average pressure exposures between different time points and arterial sites. However the actual blood pressures are known (see data in Fig A1) so why using 100 mmHg instead of the actual pressure?

We thank the reviewer for their comment. We do agree that the supine, resting brachial blood pressure of all patients was measured at all time points of the study. Indeed, this pressure was used for the calibration of the tonometer pressure waveforms. However, the supine pressures cannot be considered as the driving pressure of arterial remodelling at all phases of the study. While during HDBR supine brachial blood pressure is (approximately) representative for most arteries in the body, at baseline, the participants lived a normal life, thereby spending part of their day in a standing or seated position. Due to gravitational forces, arteries in the leg will be subjected, on average, to higher pressures than those measured brachially, while carotid pressures will be lower (please see discussion at page 19). However, an exact estimation of these pressures is not trivial, as we have no data on the average activity (i.e., body position) of a person during their normal life. Additionally, the exact vertical distance between the measurement point and the individual arteries is highly patient-specific, being dependent on height and other anthropometric factors.

For these reasons, we opted for providing a comparison of biomechanics at a common pressure of 100 mmHg, which also allows for removing the confounding factor of pressure on the biomechanical metrics. Furthermore, it is worth noting that supine brachial systolic and diastolic blood pressures varied only marginally during the study (systolic: from 112–120 mmHg; diastolic: from 66–73 mmHg).

We have restructured part of the discussion to clarify this point (Page 20, bottom half):

However, it is worth considering that we calculated circumferential stresses at a uniform pressure of 100 mmHg without accounting for expected/potential differences in average pressure exposures between different time points and arterial sites. This choice was dictated

> *by the fact that an accurate estimation of actual changes in average pressure loading at each artery would require continuous monitoring of blood pressure and body position (especially at baseline and during recovery), which was unavailable in our data. This limitation prevents more accurate evaluations of wall stress changes throughout the study. Simulated femoral and popliteal circumferential stresses likely underestimated real stresses at baseline and during recovery, which would challenge the driving role of wall stress in arterial remodelling. Indeed, it is somewhat surprising to identify comparable baseline stresses at 100 mmHg across the three arteries, given that these arteries experience, on average, very different hydrostatic pressures.*

5. the authors acknowledge that the main limitation of their approach is to neglect the adventitia in the mechanical response of arteries. However, quantitative analysis of stress distribution between media and adventitia should be provided to support this assumption, assessing how much is the fraction of stress taken by the adventitia in these arteries, otherwise all the discussion on how much stress distributes in VSMC or elastin is not acceptable.

We thank the reviewer for their comment. The assumption we made was that the intima-media thickness can be used as a surrogate measure of the total wall thickness. This assumption was necessary, given that the total wall thickness cannot be reliably measured *in vivo*. On the contrary, the measured pressure–diameter response pertains to the whole artery (i.e., including the adventitia). This implies that we systematically overestimated the stresses generated by the pressure in the artery wall, given that, in reality, the pressure load is distributed over a thicker wall. Using a tri-layered wall model, we previously estimated that in the distal thoracic aorta the adventitia bears only one third of the total pressure load at 100 mmHg of pressure and under passive (fully vasodilated) conditions (3). Notably, our current model identified a significant contribution of vascular smooth muscle cells (VSMCs) to the mechanics of the carotid, femoral, and popliteal arteries. Because VSMCs are predominantly located in the medial layers and because contraction leads to an overall reduction in circumferential stretch, the load bearing further shifts from the adventitia to the media during contraction. While we do not have any tri-layered modelling data of the arteries considered in this study, we believe our assumption that in physiological *in vivo* conditions the load is predominantly carried by the media to be reasonable.

We have clarified this point in the limitation section (Page 23, first paragraph of the limitations section):

> *Indeed, in a previous study, we estimated that the adventitia of the distal thoracic aorta bears only one third of the total pressure load at physiological pressures and in the absence of VSMC contraction (Giudici et al., 2023a). Because VSMCs are predominantly located in the medial layer, the significant contributions of VSMCs to arterial mechanics at all locations predicted by our current model would suggest a further shift of load bearing from the adventitia to the media. Therefore, while our IMT-based estimation of the wall thickness is a source of uncertainty, we believe the resulting errors on our biomechanical analyses to be relatively small.*

6. the conclusion of all the dicussion is that remodelling observed in the femoral and poplital arteries may be more driven by WSS than wall stress. The authors say they expect a reduction of WSS in the femoral and poplital arteries during HDBR and increase in recovery. Are there data supporting that?

We thank the reviewer for the important comment. Indeed, the data in the original study of Boutouyrie and colleagues (4) shows that average blood flow velocities dropped in both the femoral and popliteal artery. The concomitant decrease in the leg vessels' diameter is, therefore, indicative of a drop in flow rate. We acknowledge that this point was not sufficiently clear in the discussion. We clarified this aspect in the relevant paragraph of the discussion (Page 21, mid-page):

> *Indeed, in our cohort, blood flow velocities in the femoral and popliteal arteries decreased, on average, during HDBR (albeit not statistically significantly). This fact, together with the concomitant significant drop in diameters, is indicative of decreased local blood flows (Boutouyrie et al., 2022).*

Reviewer 3

The manuscript presents a technically well-developed and ambitious contribution addressing a longstanding challenge in arterial biomechanics: the in vivo parameterization of complex constitutive models. The authors propose a modelling framework that exploits longitudinal and multi-site data to reduce parameter identifiability issues that have so far prevented the clinical use of constrained mixture models. The formulation is rigorous, drawing from the classical foundations of finite elasticity and mixture theory, and the authors provide a detailed and transparent derivation of the constitutive equations, boundary conditions, and parameter estimation strategy.

The main strength of this work lies in its methodological innovation. The coupling of longitudinal measurements with cross-site consistency constraints is a sound and original way to improve parameter identifiability without oversimplifying the underlying physics. The application to the 60-day head-down bed rest dataset is compelling, as it allows the authors to evaluate physiological remodeling under controlled haemodynamic unloading. The quantitative agreement between model predictions and experimental data is satisfactory, with R^2 values around 0.9, and the analysis convincingly isolates the roles of smooth muscle contraction and collagen microstructural rearrangement in arterial adaptation.

The mathematical framework is clearly described and appropriately justified. The assumptions on incompressibility, thin-walled geometry, and fixed axial stretch are consistent with the physiological context. The constrained mixture model with distinct elastin, collagen, and smooth muscle contributions follows the tradition of Humphrey's and Holzapfel's formulations but introduces an elegant dimensionality reduction by imposing inter-arterial and temporal regularity. The optimization strategy is complex but carefully controlled, and the use of reduced axial force and pulse wave velocity as additional constraints is well motivated.

We thank the reviewer for their overall positive evaluation of our methodology and of the analysis of the head-down bed rest data.

However, some aspects would benefit from deeper clarification. The choice of the reference configuration, defined at mean pressure at baseline, may influence the interpretation of deposition stretches and load-bearing ratios across time; a sensitivity analysis could test the robustness of the inferred microstructural trends.

We thank the reviewer for their comment. We agree with the reviewer's assessment that our definition of a fixed reference configuration at baseline challenges the more usual definition of deposition stretches, as used, e.g., in growth and remodelling frameworks. Indeed, at any timepoint, our "deposition stretches" actually indicate the stretches that individual constituents would experience if the artery returned to its baseline reference configuration.

We clarified this point in the methods (Page 10, top half):

> *Notably, the choice of a time-independent reference configuration κ_r challenges the usual definition of "deposition stretches", as seen, e.g., in growth and remodelling frameworks (Latorre and Humphrey, 2018). In our work, the constituents' deposition stretches at each time point should be interpreted as the deformation that the individual constituents would experience if the vessel returned to the baseline reference configuration.*

However, we do not believe that our definition of deposition stretches impacted the load bearing ratios across time. Indeed, any arterial model with deposition stretches can be redefined with respect to a new reference configuration by simply applying the deformation gradient between the old and new

reference configurations to the constituents' deposition stretch matrices. This transformation would not alter the modelled responses and thus would not effect, the constituents' load bearing.

The assumption of constant constituent stiffnesses and volume fractions (except collagen) is physiologically plausible for the study duration but remains a major source of potential bias if applied to other datasets. Moreover, while the longitudinal data improve identifiability, the risk of parameter compensation among Φ and stiffness-like parameters persists, and the authors might discuss the identifiability limits more quantitatively (e.g., by reporting confidence intervals or exploring the cost-function landscape).

We agree with the reviewer that the assumption of constant constituent stiffness and, especially, elastin and VSMC volume fractions may not hold when applying our framework to longitudinal data acquired over longer periods of time. We now acknowledge this point in the discussion (Page 23, mid-page):

> *Furthermore, these assumptions on the constancy of the elastin and VSMC volume fractions should be revisited when applying our framework to longitudinal studies with longer observation times, e.g., by employing exponential decay functions to describe elastin's degradation with ageing (Latorre and Humphrey, 2018).*

Concerning the risk of parameter compensation between Φ and stiffness-like parameters, we agree with the reviewer that this aspect deserves more careful evaluation. As suggested by the reviewer, we evaluated the cost function landscape, particularly in the Φ_{VSMC}–T_{VSMC} plane. Indeed, our strategy to resolve the parameter compensation issue has been to constrain Φ_{e} based on literature values. As such, the baseline Φ_{VSMC} of the three arteries is the only volume fraction that remains freely estimated (given $\Phi_{\mathrm{c}} = 1 - \Phi_{\mathrm{e}} - \Phi_{\mathrm{VSMC}}$). As shown in **Figure R3** below, thanks to our parametrisation strategy, the cost function is convex in the Φ_{VSMC}–T_{VSMC} plane, yielding to a unique pair of optimal Φ_{VSMC}–T_{VSMC} values. In case of a parameter compensation issue, the contour plot would have shown a "vally-like" pattern, with Φ_{VSMC}–T_{VSMC} pairs in the vally equally minimising the cost function. Indeed, despite being convex, Panel A of **Figure R3** still partially retains the "valley" pattern, with errors growing less rapidly for a concomitant increase or decrease of Φ_{VSMC} and T_{VSMC}.

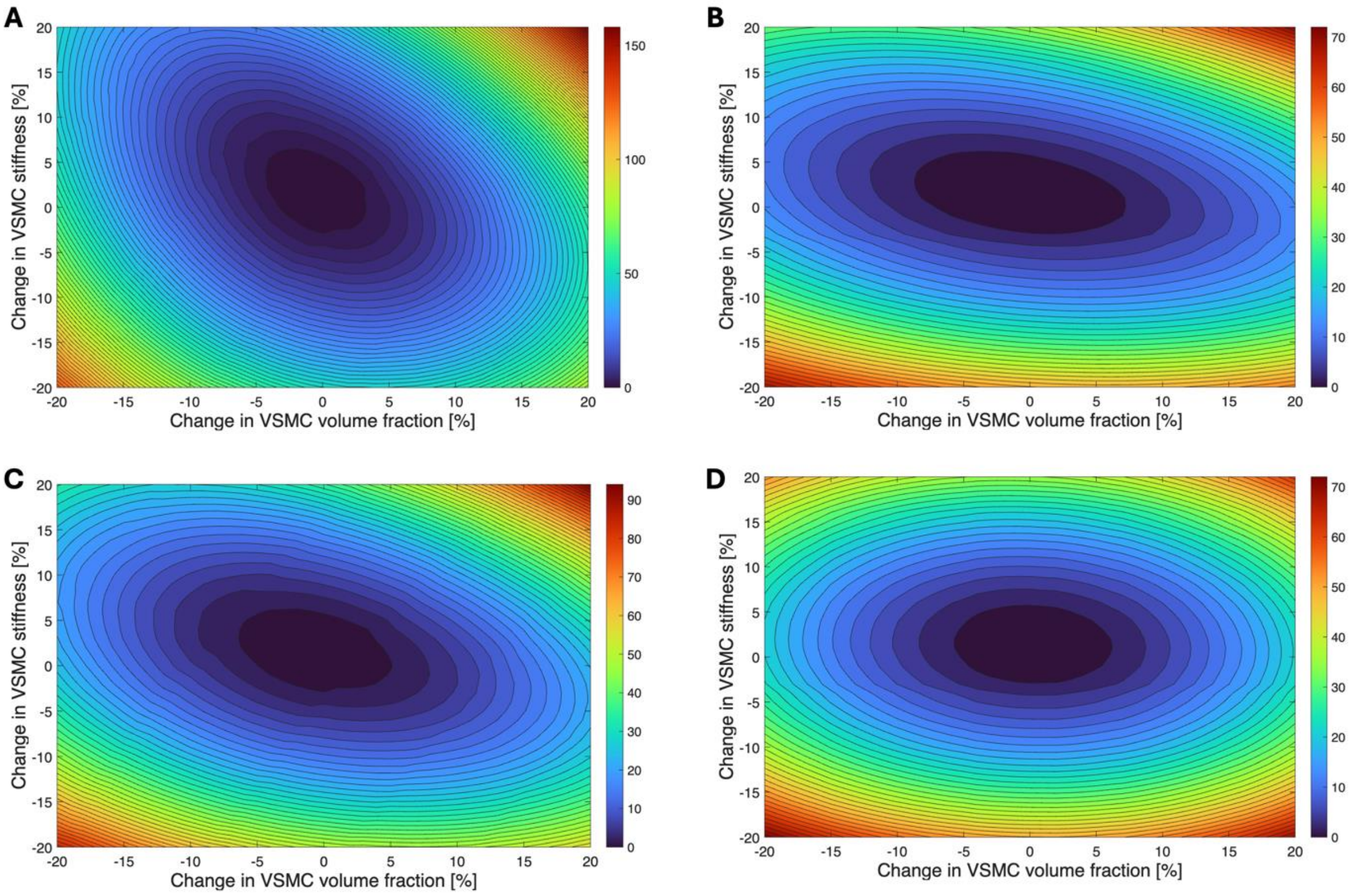

Figure R3 – Percent change in cost function value (Eq. 13) as a function of changes in VSMC volume fraction (Φ_{VSMC}) and stiffness-like parameter (T_{VSMC}). The change in VSMC volume fraction was applied uniformly on all arteries in Panel A, and individually at the carotid, femoral, or popliteal artery alone in Panels B, C, and D, respectively. Colour bars indicate the percent change in cost function value with respect to the best solution (i.e., when no change in VSMC stiffness and volume fraction was applied).

We have included **Figure R3** in the appendix as Figure A1 and referenced it in the Methods (Page 10, top):

> *Appendix **Figure A1** illustrates how this strategy ensured the convexity of the cost function in the $\Phi_{\mathrm{VSMC},t_j} - T_{\mathrm{VSMC}}$ plane.*

From a physiological standpoint, the model offers meaningful insights into vascular remodeling under microgravity analogs. The prediction of reversible peripheral de-stiffening through increased smooth muscle tone and collagen realignment is consistent with experimental evidence and mechanobiological reasoning. Yet, the discussion could more explicitly contrast the simulated wall stress homeostasis with actual pressure exposure changes during bed rest, which may not be uniform across arteries.

We thank the reviewer for their comment. We attempted to address the issue of the blood pressure exposure changes in the third paragraph of the discussion. However, as argued in response to the fourth comment of the other reviewer, the main problem is that the actual pressure exposures at the different timepoints are difficult to determine, especially at baseline and in the recovery phase. Indeed, our dataset contains brachial pressures measured supine at all time points. While these pressure are (relatively) representative of the pressure across the arterial system during head-down bed rest (except for the small hydrostatic term caused by the 6° bed inclination), supine brachial pressures at baseline and during recovery do not reflect well the average acting pressures throughout

the day, due to hydrostatic pressure changes and pressure fluctuations due to varying daily activities. One could correct brachial pressure values to account for hydrostatic effects at different arterial beds, but there would still be a large degree of uncertainty, e.g., on the relative amount of time spent by each individual in the supine, standing and seated position. Each of these positions differ in hydrostatic pressure distribution across arteries. Assuming a hypothetical person of average height and leg-to-body length ratio, and a 50:50 standing:supine split during the day yields an average ~30 and ~40 mmHg mean pressure difference between the carotid artery and the femoral and popliteal arteries, respectively.

We have clarified this point in the discussion (Page 20, bottom half):

> *However, it is worth considering that we calculated circumferential stresses at a uniform pressure of 100 mmHg without accounting for expected/potential differences in average pressure exposures between different time points and arterial sites. This choice was dictated by the fact that an accurate estimation of actual changes in average pressure loading at each artery would require continuous monitoring of blood pressure and body position (especially at baseline and during recovery), which was unavailable in our data. This limitation prevents more accurate evaluations of wall stress changes throughout the study. Simulated femoral and popliteal circumferential stresses likely underestimated real stresses at baseline and during recovery, which would challenge the driving role of wall stress in arterial remodelling. Indeed, it is somewhat surprising to identify comparable baseline stresses at 100 mmHg across the three arteries, given that these arteries experience, on average, very different hydrostatic pressures (approximately a 30 and 40 mmHg average daily mean pressure difference between the carotid and the femoral and popliteal arteries, respectively).*

Overall, the paper is technically sound, methodologically original, and physiologically relevant. It bridges a critical gap between ex vivo constitutive modelling and clinical applications. Provided that the authors clarify the treatment of the reference configuration, discuss identifiability robustness, and acknowledge the limits of parameter invariance assumptions, the work merits publication. It represents a substantial step toward quantitative phenotyping of arterial remodeling from non-invasive in vivo data.

We thank once more the reviewer for their positive overview of our manuscript and, indeed, for their constructive criticism, which we believe has contributed to improving the quality and rigour of our manuscript. We hope that our responses and additional analyses have clarified their questions and concerns.

References

1. Giudici A, van der Laan KWF, van der Bruggen MM, Parikh S, Berends E, Foulquier S, et al. Constituent-based quasi-linear viscoelasticity: a revised quasi-linear modelling framework to capture nonlinear viscoelasticity in arteries. Biomechanics and Modeling in Mechanobiology. 2023;22(5):1607-23.
2. Learoyd BM, Taylor MG. Alterations with Age in the Viscoelastic Properties of Human Arterial Walls. Circulation Research. 1966;18(3):278-92.
3. Giudici A, Spronck B, Wilkinson IB, Khir AW. Tri-layered constitutive modelling unveils functional differences between the pig ascending and lower thoracic aorta. J Mech Behav Biomed. 2023;141.
4. Boutouyrie P, Fayol A, Fortier C, Khettab H, Cristian C, Gencer U, et al. Impact of 60 days of head-down bed rest on large arteries. Journal of Hypertension. 2022;40(10).

Dear Dr Giudici,

Re: JP-RP-2025-289863R1 "**A constitutive modelling framework for applications to *in vivo* longitudinal data: Evaluation in 60-day head-down bed rest study on arterial function**" by Alessandro Giudici, Karen Barchetti, Umit Gencer, Hakim Khettab, Elie Mousseaux, Carole Leguy, Tammo Delhaas, Rosa Maria Bruno, Bart Spronck, and Pierre Boutouyrie

We are pleased to tell you that your paper has been accepted for publication in The Journal of Physiology.

The last Word (or similar) version of the manuscript provided will be used by the Production Editor to prepare your proof. When this is ready you will receive an email containing a link to Wiley's Online Proofing System. The proof should be thoroughly checked and corrected as promptly as possible.

Authors should note that it is too late at this point to offer corrections prior to proofreading. Major corrections at proof stage, such as changes to figures, will be referred to the Editors for approval before they can be incorporated. Only minor changes, such as to style and consistency, should be made at proof stage. Changes that need to be made after proof stage will usually require a formal correction notice.

All queries at proof stage should be sent to: TJP@wiley.com.

Following copyediting and typesetting the accepted version will be published online.

Yours sincerely,

Eleonora Grandi
Senior Editor
The Journal of Physiology

IMPORTANT POINTS TO NOTE FOLLOWING ACCEPTANCE OF YOUR PAPER:

• IMPORTANT NOTICE ABOUT OPEN ACCESS: To assist authors whose funding agencies mandate immediate public access to published research findings, The Journal of Physiology allows authors to pay an Open Access (OA) fee to have their papers made freely available immediately on publication.

The Corresponding Author will receive an email from Wiley with details on how to register or log-in to Wiley Authors Services where you will be able to place an order.

• You can check if your funder or institution has a Wiley Open Access Account here: https://authorservices.wiley.com/author-resources/Journal-Authors/licensing-and-open-access/open-access/author-compliance-tool.html.

• TRANSPARENT PEER REVIEW POLICY: To improve the transparency of its peer review process, The Journal of Physiology publishes online as supporting information the peer review history of all articles accepted for publication. Readers will have access to decision letters, including Editors' comments and referee reports, for each version of the manuscript as well as any author responses to peer review comments. Referees can decide whether or not they wish to be named on the peer review history document.

• You can help your research get the attention it deserves! Check out Wiley's free Promotion Guide for best-practice recommendations for promoting your work at: www.wileyauthors.com/eeo/guide. You can learn more about Wiley Editing Services which offers professional video, design, and writing services to create shareable video abstracts, infographics, conference posters, lay summaries, and research news stories for your research at: www.wileyauthors.com/eeo/promotion.

• If you would like to receive our 'Research Roundup', a monthly newsletter highlighting the cutting-edge research published in The Physiological Society's family of journals (The Journal of Physiology, Experimental Physiology, Physiological Reports, The Journal of Nutritional Physiology and The Journal of Precision Medicine: Health and Disease), please click this link, fill in your name and email address and select 'Research Roundup':
https://www.physoc.org/journals-and-media/membernews

EDITOR COMMENTS

Congratulations!

REFEREE COMMENTS

Referee #2:

Congratulations on a very nice paper, I have no further comments.
